# Computational Model of the Insect Pheromone Transduction Cascade

**DOI:** 10.1371/journal.pcbi.1000321

**Published:** 2009-03-20

**Authors:** Yuqiao Gu, Philippe Lucas, Jean-Pierre Rospars

**Affiliations:** 1 INRA, UMR 1272, Physiologie de l'Insecte: Signalisation et Communication, Versailles, France; 2 INRA, Mathématiques et Informatique Appliquées, Jouy-en-Josas, France; UFR Biomédicale de l'Université René Descartes, France

## Abstract

A biophysical model of receptor potential generation in the male moth olfactory receptor neuron is presented. It takes into account all pre-effector processes—the translocation of pheromone molecules from air to sensillum lymph, their deactivation and interaction with the receptors, and the G-protein and effector enzyme activation—and focuses on the main post-effector processes. These processes involve the production and degradation of second messengers (IP_3_ and DAG), the opening and closing of a series of ionic channels (IP_3_-gated Ca^2+^ channel, DAG-gated cationic channel, Ca^2+^-gated Cl^−^ channel, and Ca^2+^- and voltage-gated K^+^ channel), and Ca^2+^ extrusion mechanisms. The whole network is regulated by modulators (protein kinase C and Ca^2+^-calmodulin) that exert feedback inhibition on the effector and channels. The evolution in time of these linked chemical species and currents and the resulting membrane potentials in response to single pulse stimulation of various intensities were simulated. The unknown parameter values were fitted by comparison to the amplitude and temporal characteristics (rising and falling times) of the experimentally measured receptor potential at various pheromone doses. The model obtained captures the main features of the dose–response curves: the wide dynamic range of six decades with the same amplitudes as the experimental data, the short rising time, and the long falling time. It also reproduces the second messenger kinetics. It suggests that the two main types of depolarizing ionic channels play different roles at low and high pheromone concentrations; the DAG-gated cationic channel plays the major role for depolarization at low concentrations, and the Ca^2+^-gated Cl^−^ channel plays the major role for depolarization at middle and high concentrations. Several testable predictions are proposed, and future developments are discussed.

## Author Summary

All sensory neurons transduce their natural stimulus, whether a molecule, a photon, or a mechanical force, in an electrical current flowing through their sensory membrane via similar molecular and ionic mechanisms. Olfactory receptor neurons (ORNs), whose stimuli are volatile molecules, are no exception, including one of the best known: the exquisitely sensitive ORNs of male moths that detect the sexual pheromone released by conspecific females. We provide a detailed computational model of the intracellular molecular mechanisms at work in this ORN type. We describe qualitatively and quantitatively how the initial event, the interaction of pheromone molecules with specialized receptors at the ORN surface, is amplified through a sequence of linked biochemical and electrical events into a whole cell response, the receptor potential. We detail the respective roles of the upward activating reactions involving a cascade of ionic channels permeable to cations, chloride and potassium, their control by feedback inactivating mechanisms, and the central regulatory role of calcium. This computational model contributes to an integrated understanding of this signalling pathway, provides testable hypotheses, and suggests new experimental approaches.

## Introduction

**Figure 1:**
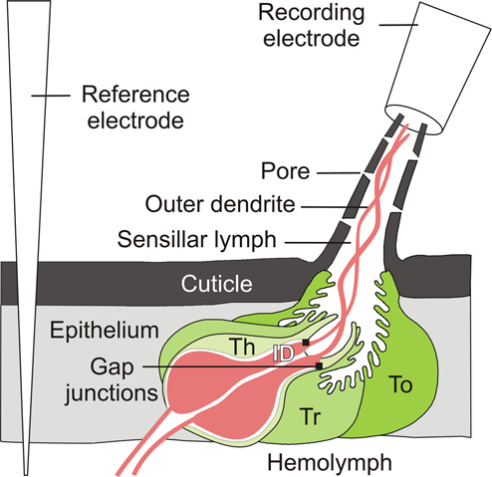
Moth pheromone-sensitive sensillum trichodeum in tip-recording conditions. The sensillum is a small organ typically composed of 2 ORNs and 3 auxiliary cells (thecogen Th, trichogen Tr and Tormogen To), housed within a porous cuticular hair. The tight junctions between cells separate the ORN extracellular environment in two parts with different ionic compositions, the sensillar lymph bathing the outer dendritic segment (sensory) and the hemolymph bathing the inner dendrite and soma. In experimental conditions the pheromone is delivered close to the hair. The ORN electrical response is recorded extracellularly with an electrode slipped on the cut hair tip. Figures 2 and 3 give detailed views of the ORN membrane processes at the molecular level. Figure 6 gives an overview of the global electrical organization of the sensillum. Modified from [8].

Olfactory receptor neurons (ORNs) are essential for the recognition of odor molecules. In vertebrates this recognition is performed by several hundreds olfactory receptor proteins (ORs) borne by the ORN plasma membrane, each ORN expressing a single type of receptor [1]. In insects a smaller number of ORs have been identified [2]–[4]. In male moths, ORNs housed in antennal sensilla trichodea (Figure 1) can detect female-released sexual pheromone with exquisite sensitivity, specificity and efficiency [5]. These ORNs have been the subject of intensive studies during the last fifty years using molecular, radiochemical, pharmacological, electrophysiological, calcium imaging, behavioral and modeling techniques (reviewed in [6]–[8]). The latter contribution has been significant and ORNs have experienced a rich history of modeling, since reports that a male moth can find a pheromone releasing female from several miles away [9],[10] and that a single pheromone molecule is sufficient to elicit an action potential in the moth sensory neurons [11]. The system has been modeled at the level of behavior [12],[13], at the level of antenna as biomechanical filter for odor molecules [14]–[16], at the level of electrical circuits that give rise to action potentials (e.g. [17],[18]), and at the level of biochemical processes that lead to neuronal activation [19]–[22]. The most detailed model yet published is that of Kaissling [23] which attempted to account for the production of the “receptor potential” through the interactions of a process generally referred to as “perireceptor events”. In fact this process consists of a biochemical network of the carrier proteins (pheromone binding proteins, PBPs), ORs and odor degrading enzymes [8],[24] which occupy a common space surrounding the outer dendritic receptive membrane of ORNs.

A reasonably complete picture of the transduction processes emerges from these studies, although some of the molecular and ionic channel mechanisms underlying the transduction process still remain elusive. After perireceptor processes, the pheromone bound ORs are believed to interact with a G-protein which in turn activates the effector enzyme phospholipase C-β (PLCβ) [25]. This enzyme catalyzes the production of second messenger molecules, inositol 1,4,5-triphosphate (IP_3_) and diacylglycerol (DAG), which trigger the opening of a cascade of various ionic channels. The resulting ionic currents generate the receptor potential (RP) which passively propagates to the ORN soma and axon where it generates action potentials. Recently, this classical metabotropic mechanism has been challenged in insect ORNs and a direct coupling of the OR to a cationic channel has been proposed in parallel or in replacement [26]–[28]. These new developments are important from molecular, physiological and evolutionary points of view.

The full description of such a complex signaling network, involving both feedforward and feedback processes, is a daunting task. Modeling can contribute to this description by integrating various effects and displaying quantitatively what results from the interplay of all molecular actors. The knowledge accumulated on the pheromonal ORN is sufficient to start building a model of its transduction cascade, and to test whether it can effectively link together some of the known facts and suggest new experiments. Thus, the first aim of our investigation was to develop a qualitative model of the pheromone transduction cascade integrating the known molecular and ionic mechanisms. The second aim was to translate these mechanisms, wherever possible, into a set of differential equations and to determine the quantitative values of their parameters. We made a systematic search of known values and determined the unknown values by fitting the model output to the properties of the experimentally measured RP. These properties were systematically determined in response to “square” pulses of pheromone of constant duration at several intensities [23],[29],[30]. They offer the most precise data on the transduction cascade available so far. These responses are characterized by a rapid rising phase, a slow falling phase, especially at high concentrations, and an extremely wide dynamic range of about 6 decades from threshold to saturation.

In insects, most modeling efforts have been dedicated to the perireceptor and receptor processes in moth pheromone sensilla [23], [31]–[35]. Although interactions of ORs, G-proteins and effectors have been recently studied [36], no model has been proposed yet for post-effector processes in insects. The model we present here focuses on these processes in male moth pheromone ORNs and takes advantage of the modeling studies available on olfactory transduction in vertebrates [37]–[42].

Beyond fitting adequately the experimental dose-response curves we addressed the following related questions. What are the functional roles of the various currents? In particular, what could be the respective roles of the direct (ionotropic) and indirect (metabotropic) gating mechanisms of the initial cationic current? What are the mechanisms behind the characteristics of the concentration-response curves (broad dynamic range, short rising time and long falling time)? What are the processes that contribute most to the amplifying function of the cascade?

## Results

In the first three subsections a formal model of pheromone transduction is presented. In the next three subsections the model is fitted to experimental data and its properties are studied.

### Qualitative Model of Pheromone Transduction

**Figure 2:**
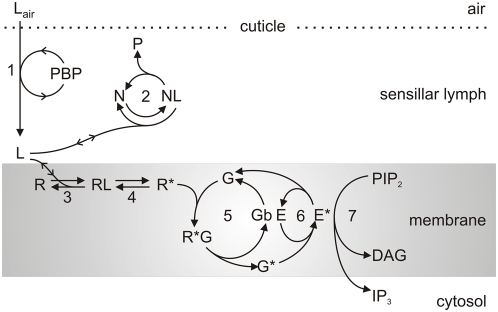
Extracellular (1–3) and early membrane (4–7) reactions involved in pheromone perireception and reception events. 1: Pheromone uptake from air (L_air_) to sensillar lymph (L) and transport through sensillar lymph by PBP. 2: Deactivation (enzyme N) producing deactivated pheromone P. 3: Interaction with receptor R. 4: Activation of receptor (R*). 5: G-protein activation (G*). 6: Effector enzyme activation (E*). 7: Production of second-messengers (DAG and IP_3_). In the present work, all these reactions were modeled as previously described [36].

**Figure 3:**
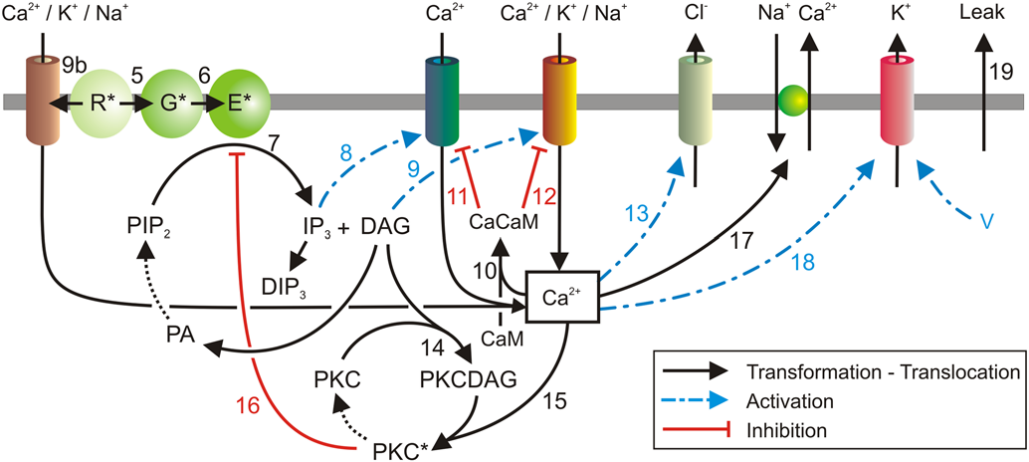
Qualitative model of membrane and cytosol reactions in moth pheromone transduction. Degradation of DAG and IP_3_, and deactivation of CaCaM and PKC* are not formally described in the present model (dotted arrows). All components are in the outer dendrite except the K^+^ channel and, possibly, the IP_3_-gated Ca^2+^ channel (see Discussion).

Based on experimental results obtained in moth ORNs, complemented when necessary with data coming from other animal species and some reasonable assumptions, we developed a global qualitative model of pheromone transduction. A schematic diagram of the model is shown in Figures 2 and 3. This model is summarized in this section. Some of the experimental results and the main assumptions (denoted A to F) on which it rests, are briefly mentioned and listed in Table 1. Complementary justifications, references and comments are provided in the Discussion section.

**Table 1:** List of main assumptions in the model.

A	Pheromone-activated receptors can bind G-proteins (metabotropic pathway) and cationic channels (ionotropic pathway).
B	CaCaM is involved in Ca^2+^ feedback of the IP_3_-dependent Ca^2+^ current.
C	Cl^−^ current is depolarizing.
D	K^+^ channels are on the inner dendritic segment.
E	Feedback inhibition of channels by CaCaM and PKC* is competitive.
F	Ca^2+^ extrusion has no antagonist.

#### Perireception (Figure 2, steps 1 and 2)

After adsorption on the cuticle the pheromone molecules enter the hair lumen through micropores in the sensillum wall. Within the aqueous sensillum lymph that fills the lumen, they bind to PBPs which carry them to the ORs borne by the ORN outer dendritic membrane. They are also degraded by enzymes [21],[43]. These processes can be fundamentally viewed as two competing effects, one which is the entrance of molecules from the outside, corresponding to an uptake measured in micromole of pheromone per liter per second, and the other which is the degradation or hypothetic deactivation [23] of pheromone molecules. When the system is stimulated by a square wave of pheromone, all pheromone molecules are not immediately removed so their concentration grows until there is an exact balance between uptake and removal. When stimulation ends, uptake returns to zero but removal continues until all pheromone molecules are removed and their concentration quickly falls to zero. This system, called flux detector by Kaissling [31], would not work without removal because the pheromone molecules are trapped inside the perireceptor space.

#### Reception and the two coupling mechanisms (Figures 2 and 3, steps 3 to 6 and 9b)

The pheromone molecule binds to an OR (step 3) then activates it (step 4), which presumably corresponds to a conformational change of the OR. In our model, the pheromone-activated OR (R*) can follow two possible pathways (assumption A). In the first pathway, it binds a G-protein to give an activated G-protein (G*, step 5) which itself combines with an effector enzyme, PLCβ [25], to produce an activated effector (denoted E*, step 6). The G-protein is involved in a loop which returns it to its initial state and the cycle can start again. The three proteins, R, G and E, can encounter one another and interact because they diffuse in the membrane. Moreover, each activated OR can activate several G-proteins when it diffuses and so contributes to signal amplification. The name of “random walk amplifier” [44] was given to this concept. In the second pathway (step 9b, top left of Figure 3), R* directly binds a cationic channel [26]–[28].

#### Second-messenger production (Figures 2 and 3, step 7)

The effector enzyme catalyzes the cleavage of phosphatidylinositol biphosphate (PIP_2_) producing IP_3_ and DAG. The respective roles of IP_3_ and DAG have not yet been completely clarified [45]–[48]. Generation of IP_3_ induced by pheromones was found to be species- and tissue-specific; it occurs only in male antennae [49],[50]. The involvement of this enzyme in insect ORN responses was demonstrated by the fast and transient production of IP_3_ after incubation of moth antennal homogenates with pheromone compounds [49],[51],[52] as well as with non-pheromonal odorants in locust and cockroach [25],[50]. Its implication has also been demonstrated by a genetic approach in *Drosophila* [53]. Upon application of pheromone, the concentration of IP_3_ increases very rapidly reaching a maximum after about 50 ms, declines quickly to a lower plateau, then declines further with a slower time course to the basal level within a few hundred ms [25]. The production of IP_3_ is GTP-dependent [25],[49].

#### Opening of calcium channels (Figure 3, step 8)

IP_3_ opens a Ca^2+^ channel. In *Manduca sexta*, Stengl [45] described a transient Ca^2+^ inward current gated by IP_3_, which declined in less than 100 ms and was inhibited by Ca^2+^-channel blockers. IP_3_-dependent ionic channels were immunolocalized in the dendritic membrane of *Bombyx mori* and *Antheraea pernyi* ORNs [54].

#### Opening of cationic channels (Figure 3, steps 9 and 9b)

First, DAG activates a non-specific cationic channel (step 9). These DAG-gated cationic channels were observed *in vivo* from outer dendritic segments in *A. polyphemus* [46] and in cultured ORNs of *Spodoptera littoralis* [48]. Also, the perfusion of sensilla trichodea with DAG increases the firing activity of ORNs in *A. polyphemus* and *B. mori* [47],[55]. Second, ORNs express an unusual member of the insect OR family, known as OR83b in *D. melanogaster* and also found in several moth species [56]. The co-expression of OR83b with conventional ORs is necessary to get odor-evoked responses both *in vivo* and *in vitro* [26],[57]. Both proteins interact with one another to form a heteromeric receptor complex [58]. OR83b, alone [27] or heteromerized with the OR [28], was recently identified as a cationic channel. So, two cationic channels are apparently involved, one which can be directly activated by R* (step 9b) and the other by DAG (step 9).

#### Closing of second-messenger-dependent channels (Figure 3, steps 10 to 12)

Ca^2+^ binds to calmodulin (CaM) to form the complex Ca^2+^-calmodulin (CaCaM) (step 10). CaCaM in turn closes the IP_3_-gated (step 11) and DAG-gated channels (step 12), hence stopping Ca^2+^ entry. First (step 11), in *M. sexta* ORNs, the IP_3_-dependent Ca^2+^ current declined quickly in normal (6 mM) extracellular Ca^2+^ concentration while it remained stable in low (10^−8^ M) extracellular Ca^2+^ concentration [45], indicating that IP_3_-dependent channels are down-regulated by Ca^2+^. Second (step 12), the amplitude of the DAG-gated current is down-regulated by CaCaM in *S. littoralis* [48]. We postulated that CaCaM is involved in the Ca^2+^ feedback of the IP_3_-dependent Ca^2+^ current as in reaction 12 (assumption B).

#### Opening and closing of Ca^2+^-dependent Cl^−^ channels (Figure 3, step 13)

An increase in intracellular Ca^2+^ activates Cl^−^ currents in moth ORNs [59]–[61]. We hypothesized that, as in vertebrates [62], the Cl^−^ current is depolarizing in insect ORNs (assumption C). No experimental evidence of the indirect inhibition of this Cl^−^ current by Ca^2+^ (for example via activated protein kinase C, PKC*) was found in *S. littoralis* [61]. For this reason we did not include any feedback regulation of this current in the basic model. However, since this is the only current without feedback, we examined a variant where it is inhibited by PKC*, in agreement with experimental data in *Xenopus* oocytes [63].

#### Feedback inhibition of PLCβ by protein kinase C (Figure 3, steps 14 to 16)

Ca^2+^ also binds to a complex of protein kinase C (PKC) and DAG. The resulting activated complex PKC* (steps 14 and 15) can phosphorylate PLC (step 16) which down-regulates its activity. In antennal homogenates from *A. polyphemus*, pheromone stimulation induces a 6-fold increase in PKC activity [55]. First, PKC, possibly activated by DAG and intracellular Ca^2+^ [64],[65], appears to be involved in the termination of the pheromone-dependent rise of IP_3_ since PKC inhibitors prolonged the pheromone-induced transient IP_3_ rise [66],[67]. Second (step 16), in many systems, a PKC-dependent feedback regulation of PLCβ has been observed [68]. In *S. littoralis* it has been shown that antennal PLCβ has PKC binding sites (Chouquet et al., in preparation). Other effects of PKC* are described below.

#### Ca^2+^ extrusion (Figure 3, step 17)

Ca^2+^ must be extruded from the ORN after stimulation. In frog [69] and squid [70] ORNs, as well as in other cell types [71], the presence of a Na^+^/Ca^2+^ exchanger (NCX) has been demonstrated. Another extrusion mechanism found in other cell types involves a PMCA (plasma membrane ATPase pump). In insect ORNs the mechanisms of Ca^2+^ extrusion are not known, which led us to compare the NCX and PMCA mechanisms.

#### Opening of Ca^2+^-dependent K^+^ channels (Figure 3, step 18)

Intracellular Ca^2+^ combined with depolarization activates K^+^ channels. The largest current in ORNs of the moths *Mamestra brassicae* and *S. littoralis* is a voltage-gated and Ca^2+^-activated current [72],[73]. This is a fast activating and sustained current with an outward rectification; K^+^ flows out resulting in membrane repolarization. The conductance of the K^+^ channels is 66 pS in *M. sexta* [74] and 180 pS in *L. migratoria* [75]. The location of these channels is unknown. In the model we assumed they are on the inner dendritic segment and soma (assumption D) because their repolarizing role is incompatible with the K^+^ concentrations on both sides of the outer dendritic membrane (see below, paragraph “Equilibrium and resting potentials”).

### Biophysical Model

The qualitative description above, although indispensable, is not sufficient to gain a proper understanding of pheromone transduction. We must now turn to a formal description of the various steps involved. Note that abbreviations in roman (e.g. G*, E*, IP_3_ etc.) denote chemical species, whereas the corresponding symbols in italics (e.g. G*, E*, *IP*_3_ etc.) denote concentrations.

#### Pre-effector steps

A formal description of the perireceptor and receptor stage (steps 1 and 2, [23],[34]) and the RGE stage (steps 3 to 6, [36]) were given previously and will not be repeated here. Briefly, it gives for a square pulse of pheromone of any duration and intensity, expressed in concentration *L*_air_ (molarity in air) or, better, in uptake *U* (mole per liter per second; *U* = *k*_i_ *L*_air_, with *k*_i_ = 10^4^ s^−1^ in the experimental conditions considered here), the concentration of the activated effector E* (and other intermediate species, including R* and G* for example) as a function of time. This system involves 13 chemical species and 12 reactions. It is described by a set of 13 ordinary differential equations and 4 conservation equations involving 17 parameters (4 initial protein concentrations, 10 reaction rate constants and 3 reaction rate constants limited by diffusion) which are given as equations (12)–(28) in the Methods section. Although very simplified, this model gives the same time-course of activated receptors as the more realistic model [23] and, likely, as the latest development of this model (Kaissling, manuscript in preparation).

The rest of this section is devoted to a formal description of the post-effector network of reactions involving diffusible modulators as well as ionic channels from which the evolution of the membrane potential can be derived.

#### Diffusible modulators

**Figure 4:**
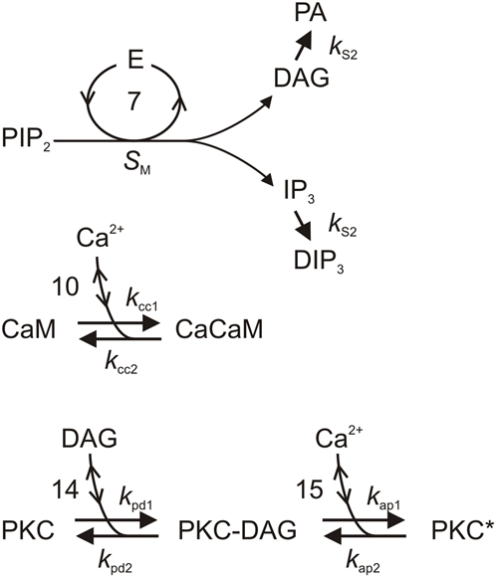
Main biochemical reactions involving diffusible molecules. The primary second messengers (DAG, IP_3_) come from their precursor (PIP_2_), the secondary messenger (Ca^2+^) comes from the sensillar lymph or intracellular stores. The two main modulators, Ca^2+^-calmodulin (CaCaM) and activated protein kinase C (PKC^*^), come from their precursors (CaM and PKC) in the presence of DAG and Ca^2+^. PA is phosphatidic acid. Reaction numbers same as in Figures 2 and 3.

The post-effector biochemical reactions involve five modulators IP_3_, DAG, Ca^2+^, CaCaM and PKC* (the latter results from the association of PKC, DAG and Ca^2+^). These reactions are depicted schematically in Figure 3 and represented in standard biochemical notation in Figure 4.

The cleavage of PIP_2_ by activated effector enzyme E^*^ producing IP_3_ and DAG is inhibited by PKC*. This is the only feedback-regulated reaction of the RGE stage in the model. The rate of production 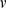 of IP_3_ and DAG was modeled by the following equation:
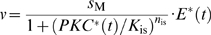
(1)where the variables are E*(*t*), the concentration of activated effector enzyme at time *t*, and PKC*(*t*), the concentration of activated PKC at time *t*. In the absence of PKC*, the reaction rate is maximal, 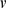 = *s*_M_ *E* ^*^, where *s*_M_ is the maximal (uninhibited) production rate. In the presence of PKC*, the other constant parameters are *K*_is_, the concentration of PKC* needed for half-maximal inhibition, i.e. 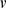 = *s*_M_ *E* ^*^/2, and *n*_is_, the Hill coefficient of the inhibitory PKC*-E* reaction.

The most important single modulator is Ca^2+^ which acts as a second messenger to open Cl^−^ and K^+^ channels and acts as an inhibitor of PLC, IP_3_- and DAG-gated channels through the CaM and PKC pathways. We considered all three initial reactants, PIP_2_, CaM and PKC, as external species, i.e. available in unlimited quantity. All reactions were modeled as standard bidirectional reactions, with a forward production and a backward degradation. Their expression as a set of first-order differential equations is straightforward, see equations (29)–(34) in Methods.

#### Ionic channels

**Figure 5:**
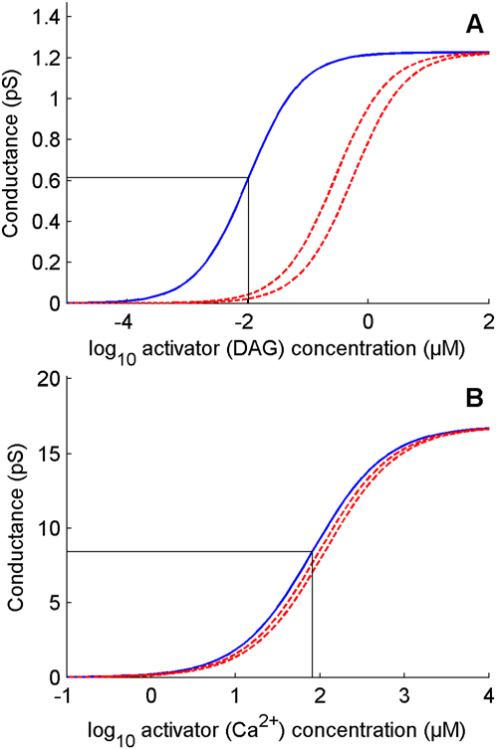
Plots of dose-conductance functions. Illustrate eqs. (2) and (3) for (A) the DAG-gated cationic conductance *G*_cat_ and (B) the Ca^2+^-gated Cl^−^ conductance *G*_Cl_. The solid blue lines in A and B represent the conductance without inhibition. The EC_50_ of the cationic current, *K*_mcat_ = 0.01 µM of DAG, is reached at *U* = 10^−4.25^ µM/s, i.e. this current is most active at low pheromone uptakes. The EC_50_ of the cationic current, *K*_mCl_ = 81 µM of Ca^2+^ corresponding to *U* = 50 µM/s, i.e. the Cl^−^ current is most active at high uptakes. The dashed red lines represent the conductance at half-maximum inhibition (intermediate curve) and maximum inhibition (rightmost curve) by CaCaM (A) and PKC* (B). The PKC* inhibition of the Cl^−^ current is very weak and practically negligible.

The ionic currents can be classified according to their gating mechanisms (molecule, ion and/or voltage) and ion permeability. The same formal description was applied to all of them, except for the OR83b cationic channel. All channels have an agonist Y, which triggers their activation, and some of them have an antagonist Z, which mediates their feedback inhibition. For example Y is DAG and Z is CaCaM for the cationic DAG-gated channel. When the concentration *Y* of the agonist increases the conductance of the channels *G*_j_ for ionic current *j* (which can be Ca^2+^, cations, Cl^−^ and K^−^) increases according to a sigmoid Hill function (Figure 5):
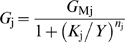
(2)where *G*_Mj_ is the maximum ionic conductance of the channels, *K*_j_ is the concentration of Y producing their half-maximal conductance, and *n*_j_ is the Hill coefficient of the agonist-channel interaction. The antagonist moves this curve to the right, i.e. decreases its sensitivity by acting on *K*_j_ (competitive inhibition, assumption E). This action involves another Hill function:
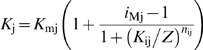
(3)In the absence of antagonist (*Z* = 0), the EC_50_ is minimum (*K*_j_ = *K*_mj_). In the presence of Z, the EC_50_ increases (*K*_j_>*K*_mj_) and the concentration-response curve *G*_j_(*Y*) is shifted to the right (see example in Figure 5A). Knowing the channel conductance *G*_j_, the equilibrium potential of the permeating ion *E*_j_ and the membrane potential *V*, the corresponding electrical current *I*_j_ is given by Ohm's law:
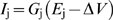
(4)where *ΔV* is the potential difference between both sides of the membrane. This is in agreement with the linearity of the experimentally measured *I*-*V* curves of the unspecific cationic [76] and Ca^2+^-gated Cl^−^ [62] channels. These equations where applied to describe the three currents having an agonist and an antagonist, i.e. Ca^2+^ current *I*_Ca_ (agonist IP_3_, antagonist CaCaM), cationic current *I*_cat_ (agonist DAG, antagonist CaCaM), Cl^−^ current *I*_Cl_ (agonist Ca^2+^).

For the Ca^2+^ extrusion current *I*_x_, in the absence of experimental data in insect ORNs, we considered both the PCMA and NCX hypotheses. The Ca^2+^ current *I*_x_ driven by the PCMA does not depend on the membrane potential. It is given by *I*_x_ = *G*_x_ *E*_x_, where *E*_x_ is the maximal pump capacity and *G*_x_ is the conductance of the pump. On the contrary, the net current *I*_x_ driven by the NCX depends on the membrane potential according to eq. (4) [77]. In both the PMCA and the NCX we assumed that the variable conductance *G*_x_ is given by eq. (2), with Ca^2+^ as agonist Y and no antagonist Z, so that *K*_x_ is a constant (assumption F).

Modified versions of these equations were used for the K^+^ and leak currents. For the K^+^ current *I*_K_ (agonist Ca^2+^, no antagonist), which is also voltage-dependent, we used a modified version of the non-inactivating Ca^2+^-dependent K^+^ current *I*_C_ [78]

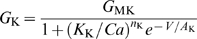
(5)where *A*_K_ is a constant and the variables are the membrane potential (*V*) and the Ca^2+^ concentration (*Ca*). Finally, the conductance *G*_ld_ of the leak current *I*_ld_ is also a constant given by the inverse of the membrane specific resistance at rest. The complete set of functions describing all 6 currents is given as equations (35)–(49) in Methods.

The OR83b cationic channel was not introduced in the present quantitative model because no formal description of its gating and regulating mechanisms is presently available. To our knowledge, no similar channel has been described in other neurons, which prevented extrapolation from known examples. The consequences of this approximation are examined in the Discussion section.

#### Receptor potential

**Figure 6:**
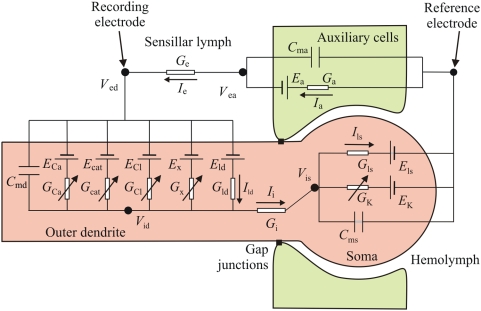
Equivalent electrical circuit of the ORN within the sensillum trichodeum (cf. Figure 1). Three main compartments are distinguished: ORN outer dendritic segment (circuit on the left with 5 conductances), ORN inner dendritic segment and soma (denoted “Soma”, circuit on the right with 2 conductances) and auxiliary cells (circuit on top with a single conductance). The experimentally recorded difference of potential (*V*_ed_) is between the sensillar lymph and the hemolymph.

If all channels were located in a patch of outer dendritic membrane and if this patch could be considered in isolation, the dynamics of the membrane potential *V*, defined as the difference of potential between inside and outside (taken as zero), would be given by:
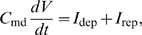
(6)where *C*_md_ is the capacitance of the membrane, *I*_dep_ is the depolarizing current
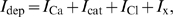
(7)and *I*_rep_ is the repolarizing current
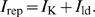
(8)However, this simple model is not applicable for two reasons. First, the difference of potential experimentally recorded is between the sensillar lymph, bathing the outer dendrite, and the hemolymph (reference electrode), bathing the inner dendrite and soma (Figure 6). These two media are separated by auxiliary cells which introduce a supplementary potential – the transepithelial potential. Second, the K^+^ channel is located in the inner dendritic segment, bathed by the hemolymph, which constitutes another compartment. Therefore, a three-compartment model distinguishing outer dendrite, inner dendrite and soma, and auxiliary cell, is needed for an adequate description of the system. Besides the potentials, leak and ionic currents described above, this introduces three new potentials (outside the outer dendrite *V*_ed_, inside the inner dendrite and soma *V*_is_, and outside the auxiliary cell *V*_ea_) and four new currents (from outer dendrite to soma *I*_i_, leak at soma *I*_ls_, through auxiliary cell *I*_a_ and along sensillar lymph *I*_e_). The functions giving these four currents are given in equations (50)–(53) and the set of differential equations linking potentials to currents is given in equations (54)–(57) which generalize equation (6) (see Methods). Potential *V*_ed_ given by eq. (55) is the most important in practice because it corresponds to the difference of potential between the recording electrode, in contact with the sensillar lymph, and the reference electrode, in contact with the hemolymph. Potentials were obtained by numerical integration of equations (54)–(57). Finally the RP was calculated as the difference of potentials between the two sides of the outer dendritic membrane Δ*V* = *V*_id_−*V*_ed_ during stimulation and at rest
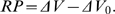
(9)However, as shown in Figure 6, the experimentally known potential is *V*_ed_, not Δ*V*. So, we computed its difference during stimulation and at rest, the so-called sensillar potential SP
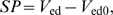
(10)(SP is nearly proportional to RP and often called “receptor potential” in the literature).

### Initial Values of the Variables and Values of Parameters

The post-effector model described above includes 65 values: the initial values of the concentrations of modulators and the membrane potential (10 values given in Table 2) and 55 parameter values. These parameters fall in 3 different categories: (1) the rate constants of the reactions involving the modulators (10 values); (2) the characteristics of the 5 main currents with 4 to 7 parameters per current (28 values); (3) the 17 parameters describing the dendritic morphology (surface and volume), the electrical properties of the dendritic membrane and the conversion factors from currents to ionic fluxes.

**Table 2:** Initial values of variables in the model.

Variable^1^	Initial Value^2^	Unit	Reference
*N*_0_	1	µM	[23], [36]
*R*_0_	1.64	µM	[23], [36]
*G*_0_	0.273	µM	[36]
*E*_0_	0.136	µM	[36]
*V*_is0_	−62	mV	[72]
*V*_id0_	−62	mV	*V*_id0_ = *V*_is0_
*V*_ea0_	35	mV	[81]
*V*_ed0_	35	mV	*V*_ed0_ = *V*_ea0_

1 In this and other tables (and in text) a dual notation is used: roman (e.g. PKC) for a species and italic (e.g. *PKC*) for its concentration.

2 For all other variables (*L*_0_, *LN*_0_, *RL*_0_, R^*^_0_, G^*^_0_, *G*_b0_, *G*_r0_, E^*^_0_, *IP*_30_, *DAG*_0_, *Ca*_0_, *CaCaM*_0_, *PKCDAG*_0_, PKC^*^_0_) the initial values were taken as zero.

However, from a practical point of view, the most important distinction is between parameters with a known value (27) and those which were unknown (38). We considered as known, and used without change, any parameter value determined in a moth ORN, especially in *Antheraea polyphemus*. If a parameter value was known in a non-moth species, especially in the frog ([40] and references therein), or as a ubiquitous component presents in any cell, we used it as a starting value. The fixed parameters based on experimental determinations or calculated from experimental data are given in Table 3. The 38 fitted parameters are given in Table 4 for modulators and Table 5 for ionic currents. Their final values were obtained as explained below and in the Methods section by comparison with experimentally known response characteristics.

**Table 3:** Fixed parameters in the model.

Parameter	Symbol	Value	Unit	Reference or Explanation
Dendritic lateral area	*S*_cut_	328	µm^2^	[103],[104]
Dendritic volume	*V*_cut_	38	µm^3^	[103]
Charge to concentration for Ca^2+^	*f*	136.37	µM.pC^−1^	*f* = 1/(*zFV*_cut_), *z* = 2 for Ca^2+^
Outer dendrite capacitance 1	*C*_md_	3.28×10^−3^	nF	[33]
Outer dendrite leak conductance	*G*_ld_	0.4373	nS	[33]
Soma capacitance	C_ms_	1.44×10^−3^	nF	[33]
Soma leak conductance	*G*_ls_	1.44	nS	[33]
Intracellular conductance	*G*_i_	2.011	nS	[33]
Sensillar lymph conductance	*G*_e_	26.77	nS	[33]
Auxiliary cell capacitance	*C*_ma_	30×10^−3^	nF	[105]
Auxiliary cell conductance	*G*_a_	3.1	nS	[105]
Equilibrium potential Ca^2+^ (outer)^2^	*E*_Ca_	140	mV	[61]
Equil. potential cations (outer)	*E*_cat_	0	mV	natural balance
Equil. potential leak (outer)	*E*_ld_	−97	mV	*E*_ld_ = *E*_ls_+*E*_a_
Equil. potential K*^+^* (inner)	*E*_K_	−62	mV	[83]
Equil. potential leak (inner)	*E*_ls_	−62	mV	*E*_ls_≈*V*_is0_
Equil. potential (auxiliary cell)^3^	*E*_a_	−35	mV	[81]

1 Conductances and capacitances of ORN and sensillar lymph were calculated for sensillum trichodeum cell A (with thick dendrite and large action potentials) of *Antheraea polyphemus* in tip-recording conditions, i. e. with cut hair tip, based on morphological data [103],[104] and electrical data [33].

2 Equilibrium potentials are given for the outer dendrite in contact with the sensillar lymph (outer) or for the inner dendrite in contact with the hemolymph (inner).

3 Gives rise to the transepithelial potential.

**Table 4:** Fitted parameters (10) of second messengers and diffusible modulators.

Species	Parameter	Symbol	Value	Unit	Sensitivity^1^	log_10_ *U* 2	Characteristic^3^
IP_3_ and DAG	Maximal synthesis rate	*s*_M_	933	s^−1^	4.73×10^−4^	−4.25	H
	IC_50_ for PKC*	*K*_is_	1.7×10^−4^	µM	4.25×10^3^	0	F
	Hill coefficient for PKC*	*n*_is_	2.3	–	−0.97	1.5	F
	Degradation rate	*k*_s2_	11.0	s^−1^	−3.74×10^−2^	−4.25	H
Ca^2+^ and Calmodulin	Ca+CaM association	*k*_cc1_	0.46	s^−1^	−0.51	−4.75	H
	CaCaM dissociation	*k*_cc2_	23	s^−1^	1.04×10^−2^	−4.0	H
Ca^2+^, DAG and PKC	PKC+DAG association	*k*_pd1_	0.21	s^−1^	−3.45	0	F
	PKCDAG dissociation	*k*_pd2_	25.0	s^−1^	3.14×10^−2^	0.25	F
	Ca+PKCDAG association	*k*_ap1_	2.27	µM^−1^ s^−1^	−0.34	0	F
	CaPKCDAG dissociation	*k*_ap2_	8	s^−1^	9.86×10^−2^	0	F

1 Greatest relative sensitivity *S*_r_ over the 26 uptakes and the three characteristics as given by eq. (60) in the Methods section with *ξ* = 10^−2^.

2 Uptake log_10_ *U* at which *S*_r_ was found (from *U* = 10^−4.75^ µM/s to 10^1.5^ µM/s per step of 10^0.25^).

3 Characteristic (H height, R half-rise time, F half-fall time) giving *S*_r_.

**Table 5:** Fitted parameters (28) of ionic channels.

Channel	Parameter	Symbol	Value	Unit	Sensitivity^1^	log_10_ *U* ^2^	Characteristic^3^
IP_3_-gated Ca^2+^ channel	Maximal conductance	*G*_MCa_	0.14	nS	−1.45	−4.75	R
	EC_50_ for IP_3_	*K*_mCa_	3.48	µM	−4.71×10^−2^	−3.5	R
	Hill coefficient for IP_3_	*n*_Ca_	1	–	−0.43	0.25	F
	Maximal inhibition	*i*_MCa_	3.08	–	−2.22×10^−2^	1.5	F
	IC_50_ for CaCaM	*K*_iCa_	0.61	µM	7.85×10^−2^	−0.25	F
	Hill coef. for CaCaM	*n*_iCa_	2.51	–	−8.53×10^−3^	−1.75	F
DAG-gated cationic channel	Maximal conductance	*G*_Mcat_	1.23	nS	1.03	0.25	F
	EC_50_ for DAG	*K*_mcat_	0.0104	µM	−93.9	0	F
	Hill coefficient for DAG	*n*_cat_	0.86	–	−2.99	0	F
	Maximal inhibition	*i*_Mcat_	53.2	–	−1.83×10^−2^	0	F
	IC_50_ for CaCaM	*K*_icat_	0.0377	µM	7.65	−4.75	H
	Hill coef. for CaCaM	*n*_icat_	0.818	–	−0.84	−4.25	R
Ca^2+^-gated Cl^−^ channel	Eq. potential Cl^−^	*E*_Cl_	−11.5	mV	6.08×10^−2^	1.5	F
	Maximal conductance	*G*_MCl_	16.8	nS	5.54×10^−2^	1.5	F
	EC_50_ for Ca^2+^	*K*_mCl_	81.2	µM	−1.10×10^−2^	0.75	F
	Hill coefficient for Ca^2+^	*n*_Cl_	1.52	–	−1.30	−2.75	H
	Maximal inhibition	*i*_MCl_	1.4	–	2.51×10^−2^	1.5	F
	IC_50_ for PKC*	*K*_iCl_	0.06	µM	−0.24	−4.5	R
	Hill coef. for PKC*	*n*_iCl_	1.1	–	2.64×10^−2^	0.75	F
Ca^2+^ extrusion	Equilibrium potential	*E*_x_	−17.1	mV	−0.12	1.5	F
	Maximal conductance	*G*_Mx_	2.21×10^−3^	nS	−658	−0.25	F
	EC_50_ for Ca^2+^	*K*_mx_	0.54	µM	0.62	−3.75	R
	Hill coefficient for Ca^2+^	*n*_x_	0.605	–	1.30	−4.75	R
Ca^2+^- and voltage-gated K^+^ channel	Maximal conductance	*G*_MK_	4.88	nS	3.40×10^−2^	−4.0	R
	EC_50_ for Ca^2+^	*K*_mK_	2.83×10^−4^	µM	−676	−4.75	F
	Coef. of voltage depend.	*A*_K_	12.5	mV	2.20×10^−2^	−4.75	F
Conversion factors Ca^2+^	For IP_3_-gated channel	*f*_Ca_	4.87	µM pC^−1^	3.56×10^−2^	−4.75	R
	For DAG-gated channel	*f*_cat_	2.50	µM pC^−1^	0.50	0.5	F

1, 2, 3 Same presentation as in Table 4.

#### Conversion factors

Some molecules (receptor, G protein, effector enzyme and DAG) are membrane bound. Their density, in molecules/µm^2^, can be expressed in intracellular concentration, in µM, using the following conversion factor
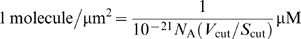
(11)where *N*_A_ is Avogadro's number; *S*_cut_ and *V*_cut_ are the lateral area and volume respectively of the outer dendrite after cutting the hair tip (see Table 3). A similar formula was used for the conversion of extracellular concentrations (with same value of *S*_cut_ but *V*_cut_ replaced with the volume of the sensillum, see Table 2 in [36]).

Ca^2+^ appears in biochemical equations as a modulator and in equations of electrical currents as a permeable ion. The latter equations describe in electrical units the movement of Ca^2+^ ions through the IP_3_-gated channels, the DAG-gated channels and the Ca^2+^ extrusion exchangers. The relationship between current *I* (in pA, i.e. pC/s) and the chemical flux *J* (in µM/s) is *J* = *fI*, where the conversion factor *f* (expressed in µM/pC) is given by *f* = 1/*zFV*_cut_ where *z* is the charge of the Ca^2+^ ion, *F* the Faraday's constant (96484×10^6^ pC·µmole^−1^) and *V*_cut_ the volume of the external dendrite (see Table 3). Different conversion factors, *f*_Ca_, *f*_cat_, and *f*_x_ were applied to the three currents. Factor *f*_Ca_ converts the inward IP_3_-gated Ca^2+^ current into a Ca^2+^ flux and takes also into account the buffering capacity of the intracellular medium. This is necessary because a large proportion of the free Ca^2+^ entering the cell (95 to 99% in adrenergic neurons; [79]) is rapidly bound to various molecules. Factor *f*_cat_ converts the DAG-activated cationic current into a flux, takes into account the buffering effect and the fact that only a fraction of the cationic current is carried by Ca^2+^ ions. Factor *f*_x_ depends on the detailed mechanism of extrusion. For the PCMA we took *f*_x_ = *f*. The NCX removes one Ca^2+^ ion for 3 Na^+^ ions. This produces a net transfer charge of one positive charge which contributes to the depolarization. Therefore the conversion factor for the Ca^2+^ flux through the NCX is *f*_x_ = 2*f*.

#### Equilibrium and resting potentials

The intracellular Ca^2+^ concentration is constrained to be smaller than 0.01 µM because Ca^2+^-dependent channels, which are closed at rest, start to be activated at this concentration [61]. The concentration of the other permeating ions is not precisely known in the outer dendrite. Na^+^ concentration was estimated at ≈1 mM and K^+^ concentration at ≈150 mM in a moth ORN [74]. For Cl^−^ no estimate was found in insects (in vertebrates its concentrations is ≈14 mM [80]).

The extracellular concentrations of these ions in the sensillum lymph bathing the outer dendrite are different from their concentrations in the hemolymph. This difference creates the transepithelial potential mentioned previously. For Na^+^ it is ≈25 mM and for K^+^ it is ≈200 mM [17],[81]. For Ca^2+^ it is in the range 1 mM [17] to 6 mM [73]. For Cl^−^ it is unknown. Reversal potentials were calculated from these concentrations: *E*_K_ is close to zero, *E*_Na_ is about 83 mV and *E*_Ca_ is about 140 mV. The reversal potentials of Cl^−^ and of the Ca^2+^ extrusion mechanism being unknown, *E*_Cl_ and *E*_x_ were fitted (see below).

As mentioned before, it follows from these values that the K^+^ channels, experimentally known to be repolarizing, cannot be located in the outer dendrite, where the equilibrium potential of K^+^ ions (≈0 mV) is too high for such a role. However, the extracellular concentrations of K^+^ in the hemolymph bathing the inner dendrite, soma and axon is 3.1 mM [82] or 20 mM [83], corresponding to an equilibrium potential *E*_K_≈−100 or −50 mV, compatible with its expected role. This is the only ionic concentration in hemolymph used in the present work. To avoid extraneous complications in modeling repolarisation, we took *E*_K_ equal to the resting potential.

The resting potential measured *in vitro* is ≈−62 mV [72],[74]. *In vivo*, the contribution of the transepithelial potential must also be taken into account. It is estimated at ≈35 mV [81]. As a result, the difference of potential at rest between the intracellular compartment of the outer dendrite and the sensillar lymph is ≈−97 mV.

### Comparison of Simulations with Experimental Measurements

Given the initial concentrations (Table 2) and the fixed parameter values (Table 3), computer simulations of the model were carried out. We searched for values of the unknown parameters, listed in Tables 4 and 5, yielding responses in accordance with experimental observations. Three sources of information were used. First, parameter values must remain in their physiological range. Second, the kinetic features of the second messengers and ionic currents must reproduce qualitatively the experimental observations. Third, the time evolution of the SP at various pheromone concentrations must agree quantitatively with the *in vivo* measurements of the SP performed in *A. polyphemus* [23],[30].

**Figure 7:**
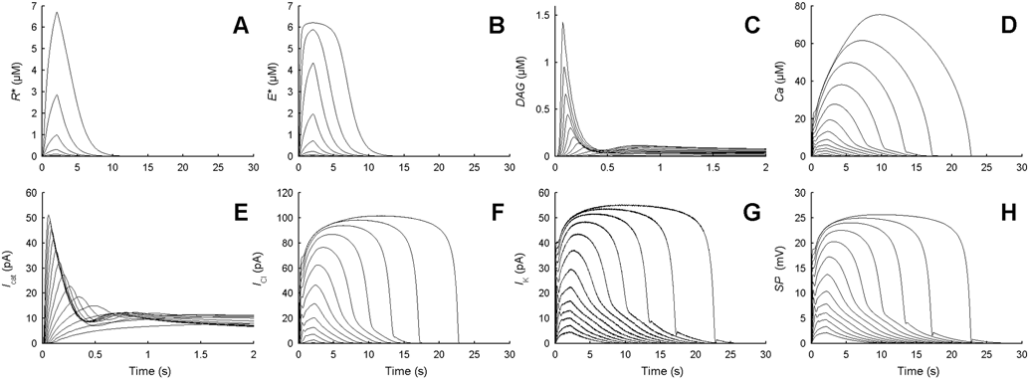
Predicted kinetics of the main chemical species, currents and potential at various uptakes. (A) Activated receptor R*. (B) Effector enzyme E*. (C) Second messengers DAG. (D) Ca^2+^. Major depolarizing currents (E) *I*_cat_ and (F) *I*_Cl_. (G) Major repolarizing current *I*_K_. (H) SP. Responses are shown for 2-s square pulses yielding different uptakes regularly spaced by 0.5 log units from 10^−4.75^ to 10^1.5^ µM/s. Note that the scales of the time axes for DAG concentration (C) and cationic current (E) are not the same as for the other species and currents. Kinetics of IP_3_ (not shown) is identical to that of DAG (C).

**Figure 8:**
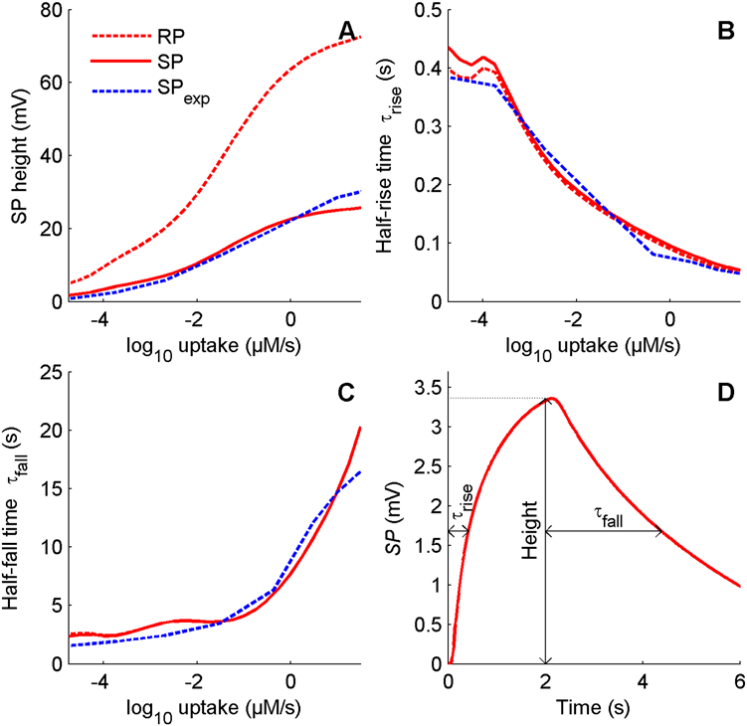
Comparison of dose-response characteristic of predicted and observed SPs. (A) Height. (B) Rising time *τ*_rise_. (C) Falling time *τ*_fall_. (D) Definition of these characteristics shown on SP response to a 2-s square pulse at pheromone uptake *U* = 10^−4^ µM/s. Characteristics of predicted SP (solid red lines) compared to those of observed SP (dashed blue lines) at 26 uptakes from 10^−4.75^ µM/s to 32 µM/s. Characteristics of predicted RP are also shown (dashed red lines). Experimental data by courtesy of K.-E. Kaissling (see [23],[30]).

Following a 2-s square pheromone pulse, the SP grows to a maximum then returns progressively to zero (Figure 7H). This simple kinetics can be summarized with three numbers (Figure 8D), its height, its rising time, measured by the time it takes to reach half maximum, and its falling time, measured by the time to fall from the end of the stimulation to half-maximum. These three quantities depend on the dose of pheromone delivered to the system, measured either as a concentration in air in micromoles per liter or better as an uptake in µM per second. Zack [30] systematically determined the amplitude, rising time and falling time of the SP at various uptakes, from threshold to saturation. On this range, the amplitude increases, the rising time decreases 10 times and the falling time increases 10 times. These three dose-response functions (Figure 8, dotted lines) were our main criteria for the fine tuning of the parameter values because they are the only ORN responses experimentally measured with precision *in vivo*.

**Figure 9:**
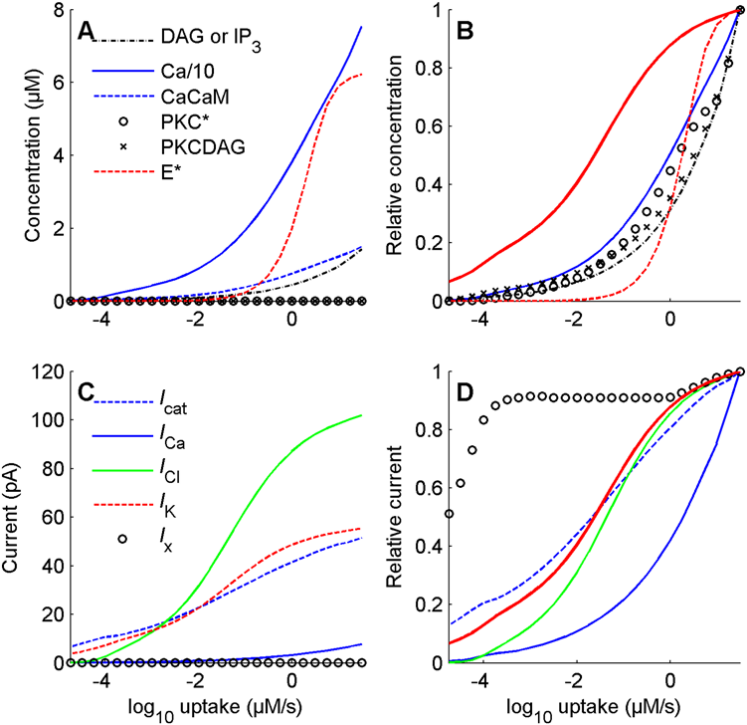
Comparison of dose-response curves for height of the chemical species and currents. Height (left column; see definition in Figure 8D) and relative height (right column) for chemical species (top row) and for depolarizing and repolarizing currents (bottom row). (A) Ca^2+^ (solid blue line) is the most abundant species (concentration divided 10 fold to be shown on the same scale as other species). (B) Responsiveness of all chemical species is much smaller than that of SP (curves shifted to the right of the SP curve shown as a solid red line), larger than that of effector enzyme E^*^ (dashed red line) at low doses and smaller than E* at high doses. (C) Cl^−^ (solid green), K^+^ (dashed red) and cationic (dashed blue) currents are the most intense currents. (D) Responsiveness of the Cl^−^ and cationic currents is higher than that of the effector enzyme (cf. (B)) and the IP_3_-gated Ca^2+^ current (solid blue). In particular, the cationic current curve is close to that of SP (solid red curve, same as in B) and K^+^ (confounded with SP) at all doses, while the curve of the Cl^−^ current (solid green) is on the right of the SP curve at low doses (smaller responsiveness) and close to it at high doses.

We modified the unknown constants in the model to fit these experimental curves by using the same stimulation conditions as used by Zack [30]. We found a set of parameter values in agreement with known facts and giving good fits (see Methods), provided the mechanism of Ca^2+^ extrusion depends on membrane potential. With the pump mechanism (PMCA), the falling time could be fitted only on a restricted range; for example if correct at low uptakes, it was much too large at higher uptakes. Therefore, all following results are given for the potential-dependent extrusion only (NCX). The values of the fitted parameters are given in Tables 4 and 5. The corresponding simulated dose-response curves are illustrated in Figure 8 (solid red lines) for the SP and in Figure 9 for the other variables (chemical species and currents).

#### Dose-response characteristic functions of the SP

Figure 8 shows that the simulated SP reproduces adequately the experimentally measured SP. First, it has the same wide pheromone stimulation range, from 10^−4.75^ to 10^1.5^ µM/s (Figure 8A solid red line). Second, it has short rising times (Figure 8B solid red line), particularly at low uptakes from 10^−4.75^ to 10^−3.75^ µM/s in which the experimental half-rise time is ca. 400 ms and only slowly decreasing when concentration increases. Third, it displays long falling times (Figure 8C solid red line), almost constant ca. 3 s from 10^−4.75^ to 10^−1.25^ µM/s, then quickly increasing from 10^−1.5^ µM/s to 10^1.5^ µM/s.

### Main Properties of the Model

With the parameter values at hand the main properties of the model can be described. Some aspects require specific attention: the kinetics of currents and chemical species, their relative importance, their relative responsiveness and the explanation of the dose-response curves.

#### Kinetics of currents and chemical species

The kinetics of activated receptor, effector enzyme, second messengers, main depolarizing and repolarizing currents, and receptor potential are shown in Figure 7. The concentration of IP_3_ (and of DAG; they are nearly identical due to the cleavage of PIP_2_) increases very rapidly and then declines quickly to a lower plateau in response to middle and high uptakes (Figure 7C). Very different types of kinetics are found. Some are phasic, others are tonic. For example, DAG production (Figure 7C) and DAG-activated cationic current *I*_cat_ (Figure 7E) are phasic, whereas Ca^2+^ increase (Figure 7D) and Ca^2+^-activated Cl^−^ current *I*_Cl_ (Figure 7F) are tonic. The phasic kinetics of *I*_cat_ results from a quick and strong inhibition by Ca^2+^ via CaCaM (Figure 5A) whereas the tonic kinetics of *I*_Cl_ results from the absence of inhibition. Interestingly, when inhibition of *I*_Cl_ by Ca^2+^ via PKC* was added to the model, a good fit with experimental SP data was obtained only for a very weak inhibition (Figure 5B).

#### Relative importance of currents and chemical species

The concentrations of the diffusible species and the intensities of the various currents are very different from one another. Their relative importance can be estimated on plots of height versus uptake (Figure 9A and 9C). Calcium is the most abundant species at all uptakes (Figure 9A). The DAG-activated cationic current *I*_cat_ is the major depolarizing current at low uptakes, and the Ca^2+^-activated Cl^−^ current *I*_Cl_ is the major depolarizing current at medium and high uptakes. Their activation at different uptakes results ultimately from the values of their EC_50_s (see legend of Figure 5). The IP_3_-activated Ca^2+^ current *I*_Ca_ is a minor current at all uptakes: its maximum conductance is small (0.13 nS, Table 5), i.e. 10 to 60 times smaller than those of *I*_cat_ and *I*_Cl_.

**Relative responsiveness of the chemical species and currents** are apparent on plots of relative amplitude versus uptake. They present significant differences. All species have a relatively low responsiveness, similar to that of the activated effector PLC, much lower than that of the SP (not shown). The EC_50_s of all species (ca. 0.5 µM/s) are much lower than that of the SP (less than 0.1 µM/s) (Figure 9B). This difference in responsiveness between species and SP can be observed at all uptakes, although it decreases at higher uptakes. The ratios of the diffusible species concentrations with respect to E* are >1 at low uptakes and <1 at high uptakes, which indicates that the dominant effect is amplification at low uptakes and inhibition of the second-messenger production at high uptakes. This dual effect is well illustrated by the curve of the IP_3_-gated calcium current (Figure 9D, solid blue line) which is close to the PLC curve (Figure 9B, dashed red) and presents the same type of responsiveness as the diffusible species. On the contrary, the cationic and Cl^−^ currents have a similar responsiveness to that of SP. Figure 9D shows that *I*_cat_ is the most sensitive current. These results mean that the DAG-gated cationic current plays a major role in depolarization at low uptakes and a minor role at high uptakes.

#### Relative contribution of currents to the SP

**Figure 10:**
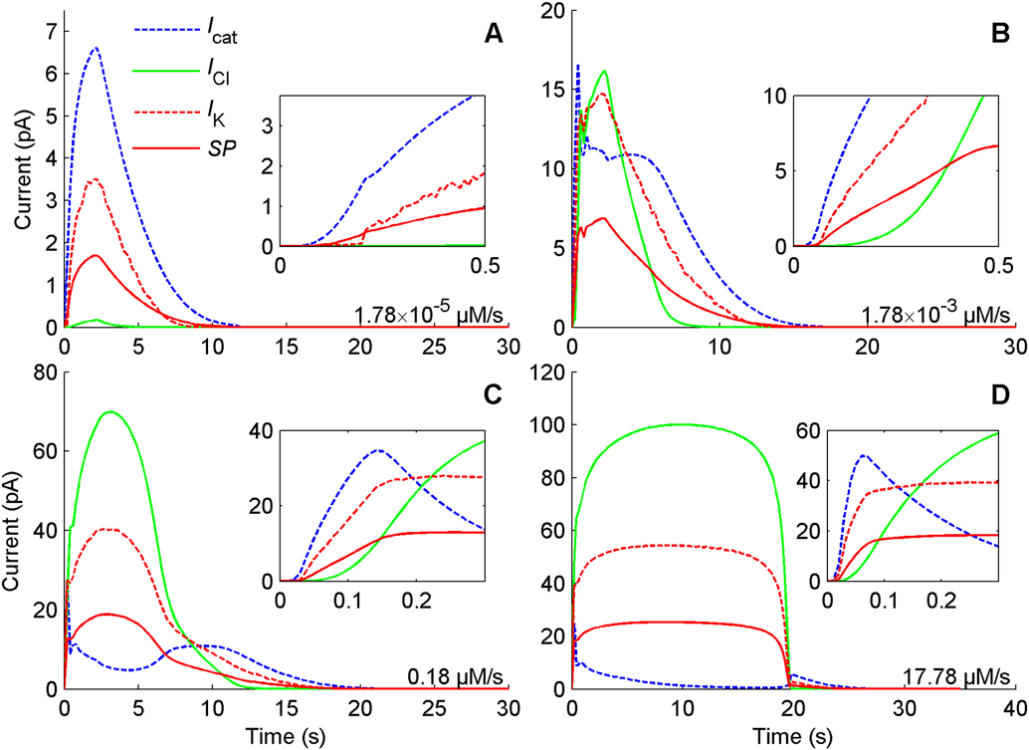
Kinetics of the major currents and SP at different pheromone uptakes. Uptakes separated by 2 log units from low to high, (A) 1.78×10^−5^, (B) 1.78×10^−3^, (C) 0.178 and (D) 17.8 µM/s. Insets show the rise of each current during the first 0.5 s (top) or 0.3 s (bottom). DAG-gated current *I*_cat_ (dashed blue) is the main depolarizing current at low dose (A). Ca^2+^-gated current *I*_Cl_ (solid green) takes over the major role at high doses (C and D). The kinetic response of the repolarizing current *I*_K_ (dashed red) is close to that of *I*_cat_ at low dose (A) and close to that of *I*_Cl_ at high doses (C and D). As shown in the insets, *I*_K_ closely follows *I*_cat_ at the beginning of the rising phase.

**Figure 11:**
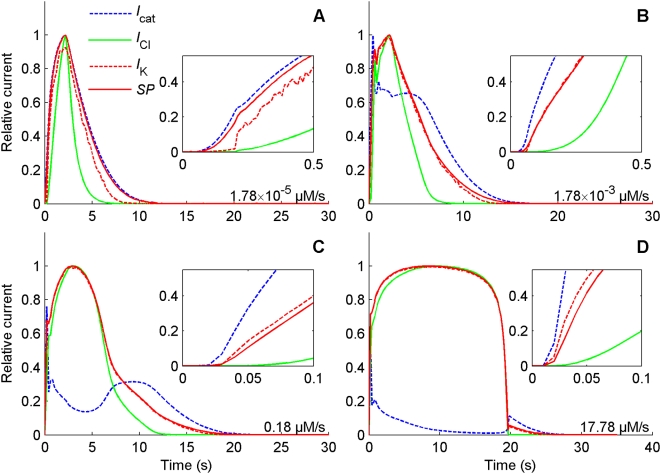
Normalized kinetics of the major currents and SP. Same as Figure 10 except that currents and SP have been normalized with respect to their maxima for easier comparison of the rising and falling phases. At all doses, (A) 1.78×10^−5^, (B) 1.78×10^−3^, (C) 0.178 and (D) 17.8 µM/s, the DAG-gated cationic current *I*_cat_ (dashed blue) rises faster than the K^+^ current *I*_K_ (dashed red) and the Cl^−^ current *I*_Cl_ (solid green), and *I*_K_ closely follows *I*_Cl_ at intermediate and high uptakes.

To analyze more precisely the relative importance of the major depolarizing (cationic and Cl^−^) and repolarizing (K^+^) currents in the generation of the SP we selected four typical uptakes at regular intervals from low to high. The kinetic of the absolute values of these currents were compared in picoampere (Figure 10) and after normalization with respect to their maxima (Figure 11). Figure 10 shows that the cationic current is the most important in both amplitude and duration at low uptakes, the Cl^−^ current takes over the dominant role at medium and high uptakes. The curve of the K^+^ current is close to that of the cationic current at low uptakes (Figure 10A) and close to the curve of Cl^−^ current at high uptakes (Figure 10C and 10D). However, the cationic current rises faster than both the Cl^−^ current and the K^+^ current (insets of Figures 10 and 11) at all uptakes. This means that the rapid rise (short half-rise time) of the SP should be attributed to the initial depolarization induced by the cationic current.

**Figure 12:**
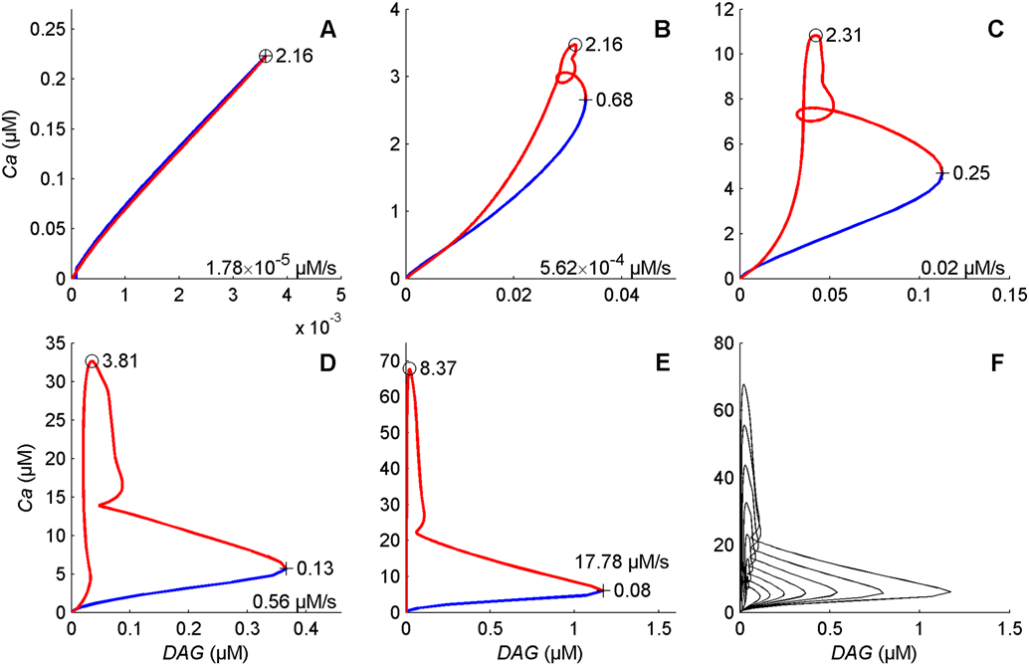
Phase portraits on the DAG-Ca^2+^ plane at different pheromone uptakes. (A) 1.78×10^−5^, (B) 5.62×10^−4^, (C) 0.02, (D) 0.56 and (E) 17.78 µM/s. (F) Superimposition of the phase portraits for 15 stimuli from low to high uptakes. Uptakes are regularly separated by 1.5 log units, i.e. multiplied by 31.6 from one portrait to the next. The starting point at *t* = 0 is close to the origin (0, 10^−3^). The blue and red lines correspond to the rising and falling phases of *DAG*, respectively. The times at which DAG (cross) and Ca^2+^ (circle) reach their respective maxima are indicated (in s). Uptakes are regularly separated by 1.5 log units, i.e. multiplied by 31.6 from one portrait to the next.

**Figure 13:**
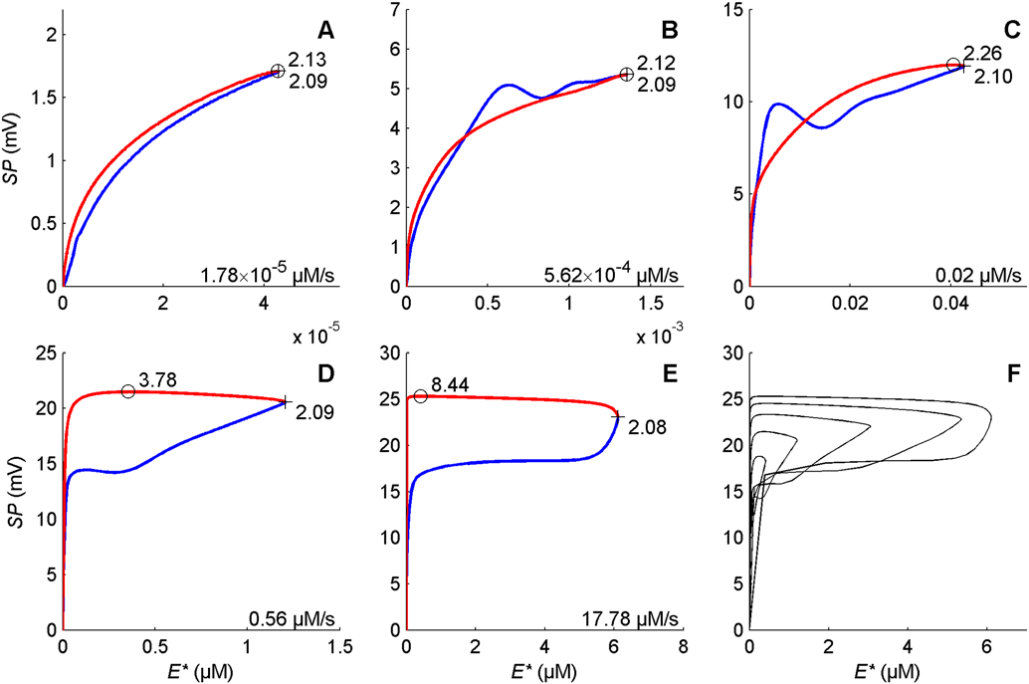
Phase portrait on the E*-SP plane at different pheromone uptakes. (A) 1.78×10^−5^, (B) 5.62×10^−4^, (C) 0.02, (D) 0.56 and (E) 17.78 µM/s. (F) Superimposition of the phase portraits. The starting point at *t* = 0 is close to the origin (0, 0). Same representation as in Figure 12.

**Phase space behavior of the modulators** presents noteworthy properties. In the model, DAG and Ca^2+^ are the two major modulators as they gate the two main depolarizing currents and activate the feedback inhibitors PKC* and CaCaM. In addition, Ca^2+^ enters mainly through DAG-gated channel (the ratio of *f*_Ca_ *I*_Ca_/*f*_ct_ *I*_cat_ very rarely exceeds 5% and its mean across all times and doses is 1.3%). The phase portraits in the DAG-Ca^2+^ plane (Figure 12) and the E^*^-SP plane (Figure 13) show how the relations between the concentrations of DAG and Ca^2+^, and between E^*^ and SP evolve in time at different uptakes. Let's consider first the DAG-Ca^2+^ relationship. At low uptakes (Figure 12A and 12B), although their values vary considerably, the ratio [Ca^2+^]/[DAG] remains approximately constant during rise and fall. The representative point of coordinates ([DAG], [Ca^2+^]) follows a closed loop in time in which the activation part (starting from the origin to the extreme point) and inactivation part (return to the origin) of the loop are practically superimposed. At higher uptakes (Figure 12C) the activation and inactivation parts start to separate, indicating a more complicated relationship. Finally (Figure 12D and 12E) there is an almost complete separation, the phase portrait taking a characteristic L-shape: high concentrations of DAG (up to 1.4 µM) are associated with small concentrations of Ca^2+^ (less than 20 µM) whereas high concentrations of Ca^2+^ (up to 90 µM) are associated with small concentrations of DAG (less than 0.2 µM). The trajectory in the phase plane shows also that the rising speed is higher than the falling speed, particularly at high uptakes, as indicated by the short times to reach the maxima of DAG and Ca^2+^ concentrations (these times are given in Figure 12). The same description holds true for the phase portrait in the E^*^-SP plane (Figure 13). At high uptakes it takes a characteristic upside-down L-shape.

#### Amplification mechanisms

A major property of the cascade is to amplify a relatively weak input signal into a strong output. This overall property can be partly quantified by the ratio *SP*_r_ /E^*^_r_ (subscript “r” stands for “relative”) of the normalized SP (output) to the normalized concentration of activated effector enzyme (input). The normalization is necessary because the two quantities are not expressed in the same units. With this definition the total amplification of the cascade depends on the pheromone uptake: it is large at low uptake then progressively decreases at higher uptakes. A noteworthy consequence of the large amplification factor at low uptakes is the leftward extension of the dynamic range. Table 6 gives the contributions of each step at uptakes 10^−4^, 10^−3^ and 10^−2^ µM/s respectively. DAG-gated and Ca^2+^-gated channels amplify the signal in different ways and they dominate the depolarization at different uptakes. At low uptakes, DAG-gated channels amplify the signal with a short rising time and the high amplification factor obtained (5300 at 10^−4^ µM/s) makes extremely weak signals detectable. At higher uptakes, IP_3_- and DAG-gated currents become transient and mainly work to provide the Ca^2+^ entry and quick initial depolarization, and the Ca^2+^-gated Cl^−^ current takes over as the dominant depolarization current.

**Table 6:** Amplification factors^1^ of each step at three different uptakes.

log_10_ *U*	IP_3_ & DAG	Ca^2+^	*I*_Ca_	*I*_cat_	*I*_Cl_	SP
−4	340	475	654	5324	670	3378
−3	74	144	134	752	322	610
−2	17	31	28	115	81	105

1 Ratios *W*_r_ /E^*^_r_, where *W*_r_ is the relative concentration of IP_3_, DAG or Ca^2+^, or relative current *I*_Ca_, *I*_cat_ or *I*_Cl_ (100% is taken at 31.62 µM/s) and E^*^_r_ the relative concentration of activated effector enzyme (100% is also taken at 31.62 µM/s).

#### Transduction delay

In order to estimate the relative contribution of extra- and intra-cellular reactions in SP rising and falling times, we stimulated directly the modeled cascade by 2-s square pulses of R* from 0.1 to 10^3^ molecule/µm^2^, instead of a square pulse of pheromone, so removing the time taken by perireception and reception processes. We found that the rising time of E* decreases from 41 to 8 ms and that of SP decreases from 32 to 18 ms. (not shown). The falling time of E* increases from 42 ms to 102 ms and that of SP increases monotonically from 0.15 s to a maximum of 3.05 s. Since the experimentally measured values of the rising time decrease from 400 to 47 ms and that of the falling time increase from 1.5 s to 17 s on the same range, this means that most of the rising (92% to 66%) and falling times (90% to 82%) result from the extracellular reactions. Moreover, as far as the intracellular reactions are concerned, the contribution of the pre-effector steps to the falling time (28% to 3%) is much smaller than that of the post-effector steps, mostly because of the slow return of the intracellular Ca^2+^ concentration at its resting level, particularly at high uptakes.

### Sensitivity Analysis of Model Parameters

The sensitivity of the system to the parameters controlling each biochemical and electrical step was analyzed as explained in the Methods section. The effects produced on the SP responses by a change in the value of a single parameter at a time were examined. The main results of this analysis can be summarized as follows (see Text S1). (i) The sensitivity of SP to the parameters depends on the characteristic (height, rising or falling time) and on the dose. (ii) Each parameter has its greatest influence on one of the characteristics (Tables 4 and 5, rightmost column). The most influenced characteristic is usually the falling time (63%). (iv) The 12 most influential parameters are *K*_is_, *G*_Mx_, *K*_mcat_, *K*_k_, *K*_icat_, *K*_pd_, *n*_cat_, *n*_Cl_, *G*_MCa_, *n*_x_, *G*_Mcat_ and *K*_iCl_. They are thus the best determined parameters. The 7 least sensitive parameters are *s*_M_, *k*_cc2_, *n*_iCa_, *i*_MCa_, *A*_k_, *K*_mCl_ and *i*_Mcat_. Tables in Text S1 list the parameters which most and least influence each characteristic.

Finally, we determined the importance of the feedback controls on second-messenger production, main ionic channels and calcium extrusion, by removing them one at a time (see Text S1). We compared the action of PKC* on PLC for different types of activation and found that it is inhibitory when activated by Ca^2+^ only, but not when activated by DAG only.

## Discussion

In this work, we propose a detailed model of the biochemical and electrical processes generating the receptor potential in the moth pheromone ORN. The role of the various currents and the molecular mechanisms of transduction are discussed in the first two subsections and the ORN response characteristics and the integrated cellular functions in the next two subsections. In the present state of knowledge, uncertainties remain on several of these processes. Therefore, the model studied is clearly incomplete and its features are not all equally well established. We discuss these uncertainties, qualitatively in the subsections “Cationic currents” and “Calcium, chloride and potassium currents” and quantitatively in the subsection “Validity of parameter values”. However, the model helps to interpret ORN properties and suggests new experiments, as discussed in the last two subsections.

### Cationic Currents

In the model the DAG-gated cationic current is the first ionic current in the transduction cascade (we attribute a different function to the parallel IP_3_-gated current, see below). A wide-spread assumption (our assumption A) is that this channel is indirectly coupled to the ORs via metabotropic pathway involving G-proteins and second messengers. This is based on the fact that like vertebrate ORs, insect ORs belong to the G-protein coupled receptor (GPCR) superfamily characterized by the canonical 7 transmembrane topology of its members [84]. However, recent bioinformatics and experimental investigations have revealed that the membrane topology of at least some *Drosophila* ORs differs from other GPCRs with an intracellular N-terminus and an extracellular C-terminus [58],[85]. The structural distinction between insect and mammalian ORs, with different membrane topology, put into question the coupling of insect ORs to G proteins [86],[87]. Recently, two studies indicated that, in expression systems, odorants can activate insect ORs and generate sensory currents independently of known G protein-coupled second messenger pathways, through a so-called ionotropic pathway [28], which may involve the cationic channel OR83b [27]. Proteins ortholog to OR83b are also expressed in moth pheromonal ORNs [56], which indicates that the ionotropic pathway may also be present in this neuron type.

However, several experimental observations made both *in vivo* and *in vitro* provide strong support for the involvement of G_q_ proteins and PLC in the moth pheromone transduction cascade. First, G_q_ proteins are present in moth antennae [88],[89] and these proteins were localized in the outer dendrite of ORNs [54]. Second, the G protein activator, NaF, activates the firing activity of ORNs both *in vivo* [89] and *in vitro* (Lucas, unpublished results), demonstrating that G_q_ proteins are functional in moth pheromone-responding ORNs. Third, *Xenopus* oocytes transfected with *B. mori* conventional pheromone receptors and without OR83b do not respond to pheromone stimuli unless they are co-transfected with G_q_ proteins [90]. Fourth, pheromone stimulation activates the PLC activity as indicated by IP_3_ production [25],[49],[50],[52].

Two other arguments, obtained from non-pheromonal ORNs, strengthen the observations above in pheromonal ORNs. First, in addition to the fast and transient ionotropic pathway, at low concentration odorants also activate G proteins and the production of second messengers in expression systems [27]. This metabotropic current develops after a longer latency and with a slower kinetics but is more sensitive to odorants than the ionotropic pathway. Second, strong genetic evidence supports a role for a Gq- and PLCβ-mediated signaling cascade during olfactory transduction in *Drosophila* [53].

The results at hand suggest the coexistence of two signaling pathways in both pheromonal and non-pheromonal ORNs, one ionotropic, the other metabotropic. Remarkably, both pathways gate a cationic channel, although it is not known whether the cationic channels of both pathways are the same (OR83b) or not, and if different whether their conductances are the same. The ionotropic signal, not amplified, is rapid and transient, and the metabotropic, amplified, is sensitive and prolonged, the former being more visible at high odorant concentration and the latter at low concentration. So, the relative intensities and timings of the two currents might be significantly different. If these properties are confirmed in future studies, they would suggest that the intensity of the cationic current found in the present model should not be entirely attributed to the DAG-gated channel because part of it comes from the receptor-gated channel. An advantage of the ionotropic pathway, especially at high uptake when the number of activated receptor is the largest and the ionotropic current is expected to become significant, is that its energetic cost for the neuron is lower. According to this view one might expect that the G-protein pathway is more inhibited at high uptake than found in the present model. The suppressed current would be compensated by the energy-saving direct coupling mechanism and the global effect would be the same. At the present time, the relative contribution of the two channels cannot be specified, if only because the reaction rate of R* with OR83b is not known. A second advantage of the OR83b cationic current is its time course. The receptor-gated current is expected to appear first and therefore to trigger an action potential faster than the DAG-gated current. The ionotropic pathway could contribute to explain the high speed of response of ORNs, especially at high pheromone concentration, and the very fast behavioral response of moths, down to 150 ms, both to contact and loss of pheromone filaments during their oriented flight to calling females [91]. The latency of the initial response was not considered in the present study and deserves more attention in the future.

### Calcium, Chloride, and Potassium Currents

It remains uncertain whether the role of the Cl^−^ current is depolarizing or repolarizing, because the intracellular and sensillar concentrations of Cl^−^ are not known. We assumed that this current is depolarizing (assumption C) for three reasons. First, in vertebrates, the Cl^−^ equilibrium potential is more positive than the resting potential [92] and a similar Ca^2+^-activated Cl^−^ current amplifies ORN depolarization ([93]–[96], see review [97]). Second, insect PBPs present in the mM range provide organic anions to the sensillum lymph [8] which decreases the sensillar Cl^−^ concentration due to the principle of charge neutrality. Third, pheromone responses (SP and action potential firing) recorded *in vivo* were significantly higher when the sensillar Cl^−^ concentration was lowered from 215 to 18 mM (Lucas, unpublished results).

One of the main uncertainties on channels (and other proteins) concerns their exact spatial location, which entails uncertainty on the mechanisms of Ca^2+^ entry. Because the present model depends only on time, any diffusion or translocation is expressed in temporal (not spatial) terms and reflected in the reaction constants. This simplification has generally no incidence, except for two channel types. The first one relates to IP_3_-gated channels which have been located in the outer dendritic membrane, based on electrophysiology [45] and immunocytochemistry [54]. This location implies an inward flow of Ca^2+^ from the sensillum lymph. However, besides or in replacement of this dendritic membrane channel, channels located in the endoplasmic reticulum may be considered. In this case Ca^2+^ would come from intracellular stores. However this hypothesis is weakened by the fact that no intracellular Ca^2+^ stores have been found in the outer dendrite. In fact the present model is compatible with both possibilities and cannot discriminate them. A third possibility is discussed in the next paragraph.

The second channel for which the spatial localization is important is the repolarizing K^+^ channel. *A priori* it can be located either in the outer dendrite or in the inner dendrite and soma region (as we did, our assumption D). The main objection against its outer dendritic location is that the equilibrium potential there is close to 0 mV, so that K^+^ cannot have a repolarizing function. Against the inner dendritic location one can mention their modulation by Ca^2+^ [72],[74],[75] because Ca^2+^ would have to diffuse from the outer to the inner membrane to trigger them which is unlikely due to the poor diffusive ability of this ion. A possible solution to this problem is that IP_3_-gated channels are in the inner dendrite. The IP_3_ synthesized in the outer dendrite would have to diffuse to the inner dendrite and soma, which is compatible with the diffusive property of this second messenger, and, there, it would gate the Ca^2+^ channel, possibly from Ca^2+^ stores. Ca^2+^ and voltage would then trigger the repolarizing K^+^ current with a delay due to IP_3_ diffusion.

This distinguishes the functions of these two different channel types in the model. IP_3_-gated channels let Ca^2+^ ions flow into the cytoplasm, whereas DAG-gated channels also depolarize the ORN at low uptakes (see below). In fact it can be shown in the model that the IP_3_ pathway is unnecessary for the depolarization.

On the basis of available experimental data we have included a feedback regulation of IP_3_ channels (via CaCaM, our assumption B), but not of Cl^−^ channels (via PKC^*^). However, in the latter case, we studied the hypothesis of a feedback inhibition via PKC* [63]. We found that both regulations are not essential (Figure 5B). For reducing the computation time we ignored the known feedback regulations on the pre-effector steps [66],[98],[99]. They are likely important for repetitive pheromone pulses that occur in natural conditions. In single-pulse condition, as in this study, this simplification presents no inconvenience provided the inhibitory regulation of, say, the receptors is not much faster than that of the effector enzymes.

All actors involved in the model have been shown to exist in the ORN membrane. The only exception is the Ca^2+^ extrusion pathway which has not yet been described in moth ORNs, although it plays an essential role in the falling phase of SP. Because of the poor intracellular diffusion of Ca^2+^ ions, it is likely that these mechanisms are located in the outer dendrite. We have shown that only a voltage-dependent extrusion is compatible with the experimental data, which strengthens the hypothesis of a Na^+^-Ca^2+^ exchange (NCX), without ruling out the presence of an ATPase pump (PMCA). We found that the NCX pump needs no negative feedback control and that its reversal potential is *E*_x_≈−17 mV. Knowing the reversal potential of Ca^2+^ (*E*_Ca_≈140 mV) and the relationship between *E*_x_, *E*_Ca_ and *E*_Na_, see eq. (46) in Methods, this value of *E*_x_ implies *E*_Na_≈88 mV, which is a reasonable value. Further experimental and theoretical investigations are needed to clarify the Ca^2+^ extrusion mechanisms.

For simplicity we have not included the Ca^2+^-gated Ca^2+^-permeable cationic channels described in *M. sexta* ORNs [100] because with Ca^2+^ entering the ORN and at the same time gating the channel, the control of the falling phase of the SP is made extremely difficult. Moreover, Ca^2+^-activated cationic channel could not be found in *S. littoralis* ORNs [61]. Perhaps qualitative differences exist in ion channel expression in ORNs across moth species providing different voltage- and time-dependent down-regulation mechanisms.

Finally, when stimulated repetitively or for a longer time the ORN adapts and its response characteristics are different from those analyzed here [30]. Adaptation is beyond the scope of the present work as it may involve reactions other than those built in the present model.

### Validity of Parameter Values

With the parameter values given in Tables 4 and 5 the model obtained accounts for several experimentally known properties of pheromone transduction in moths:

The predicted SP reaches a maximum value and follows a time course, both in its rising and falling phases, that quantitatively agrees with the measured characteristics (depolarization, rising time and falling time) of the SP as a function of pheromone uptake (Figure 8).

The model also accounts quantitatively for the transient course of IP_3_ production, as described in stop-flow experiments [25], with a very rapid increase followed by a quick decline at middle and high uptakes (Figure 7C). It follows that the kinetics of DAG and the currents gated by both IP_3_ and DAG must also be transient in this range of uptakes, which is the case in the model (Figure 7E). The transient course of second messengers at middle and high uptakes results from a strong feedback inhibition. At low uptake the inhibition is much weaker and thus the course is not transient.

On the contrary, the intracellular Ca^2+^ concentration (Figure 7D) and the Ca^2+^-gated Cl^−^ current (Figure 7F) become sustained in the range of uptakes where the second messengers and their gated currents become transient: they increase at a slow rate then decrease gradually. These features are in agreement with experimental findings. Moreover, this Cl^−^ current (*I*_Cl_) is the major component of the depolarizing currents at middle and high uptakes, which qualitatively agrees with experimental data in the frog [101].

The phase portraits E*-*SP* (Figure 13) show that at most uptakes except the highest, the maximum of E* and SP are reached at the same time and the trajectories of the rising and falling phases are close. This is equivalent to the fact, described by [23] that the concentration *R*_1_* giving a certain value of SP during the rising phase and the concentration *R*_2_* giving the same value of SP during the falling phase are equal, except at high uptake.

The experimental variability of SP is the highest at high uptake [30]. This can be interpreted, in the framework of our model, because we have shown that relatively small changes in some parameter values in different ORNs can lead to relatively large SP changes in this range of uptakes (Table 5).

However, several limitations affect the determination of the parameter values. Besides qualitative limitation regarding the completeness of the model, two other kinds of limitations must be taken into account.

First, the model is based on ordinary differential equations depending only on time which entails two limitations: space is neglected, as discussed above, and very small concentrations are not adequately described because all chemical species must be in sufficiently large number to be considered as continuous variables. The total number of activated receptors per ORN is 30 when the uptake is 10^−2.5^ µM/s [36]. The bottleneck of the whole cascade being the receptors, all uptakes greater than this can be considered as adequately described by ordinary differential equations. Presently, for uptakes less than 10^−2.5^ µM/s, only mean values are obtained. A complete description in this range will require a stochastic approach, at least at the receptor level.

Second, supposing the model qualitatively valid for the stimulus used, the problem of the precision of the parameter estimation arises. The sensitivity analysis we performed permitted a classification in two categories (see tables in Text S1): the parameters whose modification changes significantly the SP response (right columns), which were therefore estimated with good precision, and those which do not influence much the SP (left columns), which are less well estimated. For example the equilibrium potentials need not be precisely known. This analysis one parameter at a time gives only a partial view because some parameters are linked. For example, the concentration E* and the maximum synthetic rate of *s*_M_ of activated effector appear as a product, see equations (29) and (30) in Methods, so they cannot be known independently. The value given for *s*_M_ is valid under the assumption that *E*_0_^*^ = 0.136 µM [36]. However, it must be realized that the problem of parameter estimation is extremely constrained because the range of acceptable values of most parameters is restricted and because of the many feedback reactions. As a consequence the important parameters are not the same at all uptakes, especially for falling time. For this reason, finding a solution that works at all uptakes proved very difficult and suggests that a significantly different set of parameters in agreement with the experimental data available will not be easy to find.

### Interpretation of the Global ORN Properties

The present model helps to interpret global properties of the ORN, especially its performance at efficiently encoding the stimulus. The response characteristics (amplitude, rising and falling times) of the SP in the pheromonal ORN present three remarkable properties: a wide dynamic range, a short rising time which decreases with pheromone concentration, and a long falling time which increases with concentration. The proposed model explains all these features. They can be analyzed from two different points of view: the relative contribution of the pre- and post-effector steps and the mechanisms by which the post-effector steps contribute to the observed features. (1) First of all, the post-effector cascade contributes considerably to the *large dynamic range* which extends over about 6 decades from 10^−4.75^ to 10^1.5^ µM/s. Indeed, the dynamic range of the effector response is 3.25 decades only. The post-effector extension from 3.25 to 6 decades results from the collaboration of the two main currents, the DAG-gated cationic current and the Ca^2+^-gated Cl^−^ current, which present a large difference in responsiveness. The cationic current has an (uninhibited) efficient concentration EC_50_ of 0.01 µM of DAG, which is reached at a pheromone uptake of 10^−4.25^ µM/s (Figure 5A), whereas the Cl^−^ current has an EC_50_ of 81 µM of Ca^2+^, which would be reached at an uptake of 50 µM/s (Figure 5B), a value not actually reachable because the perireception system saturates at ≈30 µM/s. This means that the cationic current is most active at low uptakes whereas the Cl^−^ current is most active at high uptakes. They complement one another and contribute, by the separation of their EC_50_s, to widen the dynamic range of the ORN. (2) The *fast rising time* of the SP results from the cationic current because at all uptakes it is faster than the Cl^−^ current (compare the dashed and solid curves of insets in Figure 10). (3) The *long falling phase* of the SP is also explained by the two main currents. It reflects primarily the time course of the cationic current at low uptakes and the time course of the Cl^−^ current (which closely follows the time variation of intracellular Ca^2+^) at high uptakes. The long persistence of Ca^2+^ at high uptakes suggests that Ca^2+^ extrusion is not fast enough. However, as shown by the direct stimulation of receptors in the model, the bottleneck which limits the speed of rise and fall of SP is in the extracellular processes. Although involved and with many steps, the intracellular processes, as modeled here, are very fast, which confirms Kaissling's analyses [23].

An important function of the cascade is to transform a weak initial signal (pheromone binding to OR) into a strong local signal (RP and its corresponding SP). As already shown in a previous work [36] the amplification provided by the pre-effector stage, as quantified by the ratio *E* ^*^_r_/*R* ^*^_r_, is relatively small at any uptake (always less than 7.5). Consequently most of the amplification is provided by the post-effector stage, as quantified by *SP*_r_/*E* ^*^_r_ (Table 6). The main amplification is in the electrical stages (*I*_cat_ and *I*_Cl_), since the amplifications of all chemical stages are relatively small. Cationic and Cl^−^ channels amplify the signal in different ways and they dominate the depolarization in response to different uptakes. Cationic channels amplify the signal at low uptakes with a short rising time. Cl^−^ channels amplify the signal by a larger maximal conductance and a longer duration of depolarization. The high amplification factor at low uptakes involves several mechanisms acting inversely on the activation and inactivation processes. Moreover these mechanisms are not the same at different uptakes. At extremely low stimulation uptakes, inactivation processes have weaker effects. This can be partly explained by the value of Hill coefficients which is ≤1 for *n*_Ca_, *n*_cat_, *n*_x_, and *n*_icat_ but >1 for *n*_is_ and *n*_iCa_. This means that the inhibition of the production of second messenger (IP_3_ and DAG) and of the IP_3_-gated conductance develops on a relatively narrow range of concentration of their modulator, i.e. changes relatively abruptly in a threshold-like manner. On the contrary, the activation of the IP_3_- and DAG-dependent conductances and Ca^2+^ exchangers develops on a wider range of concentrations, i.e. more smoothly.

### Perspectives

The field of insect olfactory transduction has generated an ever increasing amount of data but dispersed and fragmentary. The complication and sometimes confusion that result from these circumstances justify an attempt to unite the parts in a comprehensive view and formal synthesis of how things might work. However, because it is clearly incomplete and involves several assumptions, the model presented here should not be considered uncritically as a faithful description of reality. Like any model, it is intended primarily as a starting point for addressing new questions, designing new experiments, and offering a tentative framework for their interpretation.

This model calls for three kinds of experiments. First, experiments on specific components. For example, are the contributions of the ionotropic and metabotropic pathways to olfactory transduction in agreement with our tentative proposal? Is the membrane repolarized by a K^+^ channel depending on an IP_3_-gated Ca^2+^ channel, both located in the inner dendrite as suggested here? What is the exact mechanism of Ca^2+^ extrusion? Second, can the model be extended to account for adapted or periodically stimulated neurons? Can it be induced to oscillate like in mammals [41],[42],[102]. Finally, can qualitative differences in all these mechanisms be found between different ORN types in the same and different biological species? All these experiments have the potential to confirm or invalidate the views presented here and so to yield significant progress in our integrated view of the ORN functioning.

## Methods

### Model Equations

#### Differential equations for pre-effector events

These equations describe the uptake, perireception, reception and early amplification in the moth pheromone receptor neuron. The species and reactions are defined in Figure 2. The values of rate constants are given in [36]. The initial values of the species are given in Table 2.
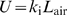
(12)

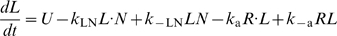
(13)

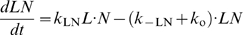
(14)

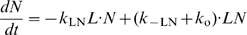
(15)

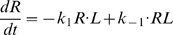
(16)

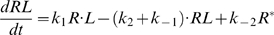
(17)

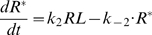
(18)

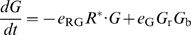
(19)

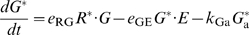
(20)

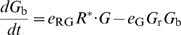
(21)

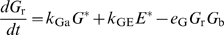
(22)

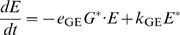
(23)

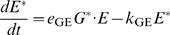
(24)


The corresponding conservation equations are:
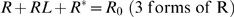
(25)

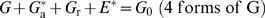
(26)

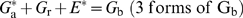
(27)

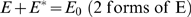
(28)


#### Differential equations for post-effector diffusible species

They describe the biochemical reactions of IP_3_, DAG, Ca^2+^, PKCDAG, PKC* and CaCaM. The reaction rate constants are defined in Figure 4. The initial values of the diffusible species are given in Table 2. No conservation equations were used for these species.
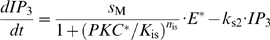
(29)

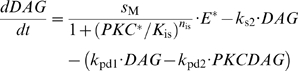
(30)

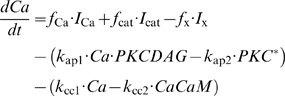
(31)

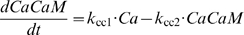
(32)

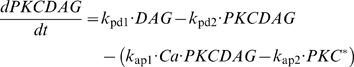
(33)

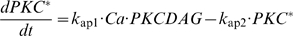
(34)


#### Functions for currents

Currents described by (35) to (47) are in the outer dendritic segment. The other currents are in the other parts of the sensillum (inner dendritic segment, soma, auxiliary cells, sensillar lymph). Currents, potentials, conductances, capacitances and batteries are shown in Figure 6.

IP_3_-gated Ca^2+^ current *I*_Ca_

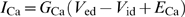
(35)

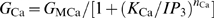
(36)

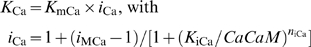
(37)


DAG-gated cationic current *I*_cat_

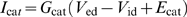
(38)

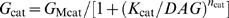
(39)

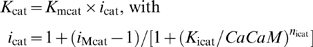
(40)


Ca^2+^-gated Cl^−^ current *I*_Cl_

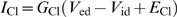
(41)

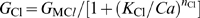
(42)

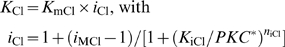
(43)


Na^+^/Ca^2+^ exchange current *I*_x_

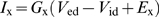
(44)

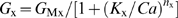
(45)

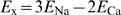
(46)


Leak current *I*_ld_ at outer dendrite
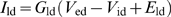
(47)


K^+^ current *I*_K_ at inner dendrite and soma
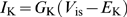
(48)

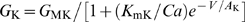
(49)


Leak current *I*_ls_ at inner dendrite and soma
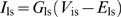
(50)


Longitudinal currents from outer dendrite to soma, in auxiliary cell and in sensillar lymph
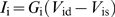
(51)

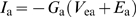
(52)

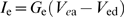
(53)


#### Differential equations for potentials

Potentials inside (*V*_id_) and outside (*V*_ed_) the outer dendrite, inside the inner dendrite and soma (*V*_is_), and outside the auxiliary cell (*V*_ea_), see Figure 6.
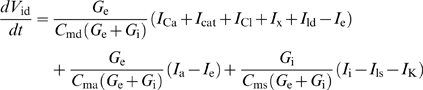
(54)

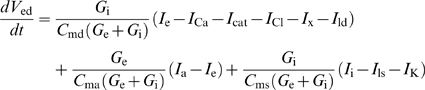
(55)

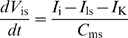
(56)

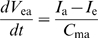
(57)


#### Numerical integration

The system of differential equations given above was integrated with the Matlab ode45 solver (The MathWorks, Natick, USA).

### Parameter Estimation

The unknown parameters of the model were estimated by utilizing various search methods based on the following criteria. First, we imposed that each parameter be in a physiologically acceptable range of values compatible with the properties of our qualitative model of transduction: the order of magnitude of Hill coefficients is one; the IP_3_-gated channel is permeable to Ca^2+^ only while the DAG-gated channel is permeable to Ca^2+^ and other cations, so that *f*_Ca_>*f*_cat_; the reversal potential of the Cl^−^ channel *E*_Cl_ must be more positive than −97 mV to be depolarizing. Second, we considered a parameter set as acceptable if the predicted kinetics of the sensillar potential were close to the experimentally measured kinetics at all uptakes. For checking this condition, we minimized a cost function based on the three response characteristics, height (*H_i_*), rising time (*τ*_rise,*i*_) and falling time (*τ*_fall,*i*_) at a series of uptakes *i* for which these characteristics were determined experimentally. The differences, *ΔH_i_ = |H_i_*−*Ĥ_i_|*, between the values *Ĥ_i_* predicted by the model for a given set *θ* of parameter values and the experimental values *H_i_*, were determined at every uptake *i*. The differences *Δτ*_rise,*i*_ and *Δτ*_fall,*i*_ were determined in the same way. Because the three characteristics vary on different scales, the differences were weighted and summed to produce a single cost function
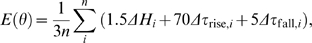
(58)where *n* = 26 is the number of uptakes. Third, a solution was finally accepted only if it was in qualitative accordance with other available experimental facts: the transient feature of the kinetics of the second messengers and IP_3_-gated currents, the sustained property of the Cl^−^ currents and K^+^ currents, and the condition that intracellular Ca^2+^ concentration must not exceed 200 µM.

Two search methods were utilized in sequence to find the parameter values. First, for a global exploration of the parameter space, we relied on a trial-and-error method. We compared a few thousands parameter sets, drawn from sets *θ* obeying the first criterion above, at 3 uptakes (low, medium, high). Most sets led to unacceptable cost functions *E*(*θ*) and were rejected. The best sets were further selected on the third criterion then tested at more uptakes. Eight presumptive solutions tested at all uptakes with *E*(*θ*) in the range 2.74–5.38 were found fulfilling the three criteria. Second, the best presumptive solution for which *E*(*θ*) = 2.74 was locally optimized utilizing the Matlab unconstrained minimizer fminsearch based on the Nelder-Mead simplex (direct search) method. The algorithm converged on the set of estimated parameters *θ*_0_ shown in Tables 4 and 5. With these parameter values *E*(*θ*_0_) = 2.61.

### Sensitivity Analysis

The sensitivity of a model response *M* to a single parameter *b_i_* can be expressed as a sensitivity function 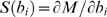. This partial derivative was estimated as the central finite difference (59) using both the forward and backward differences
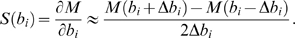
(59)This equation is only valid for an infinitesimal variation (perturbation) of *b_i_* (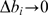). Practically, Δ*b_i_* was implemented as the product Δ*b_i_* = *ξb_i_*, where *b_i_* is the nominal parameter value as estimated above and *ξ* is a perturbation factor. We took *ξ* = 0.01, large enough to avoid numerical inaccuracies and small enough to prevent the nonlinearity of the model to play a role in the sensitivity calculations. In our case, the model responses *M_u_* are the height, half-rise time and half-fall time at different uptakes log_10_ *U* (denoted here as subscript *u*), which have different units and take values of different orders of magnitudes. In order to compare their sensitivities *S_u_*(*b_i_*) we normalized them by the model response *M_u_*

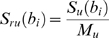
(60)For each fitted parameter, the normalized sensitivities *S_ru_* of the 3 characteristics were calculated at 26 values of *U* from threshold to saturation. The characteristic and the uptake giving the largest absolute value *S_r_* (*b_i_*) of the *S_ru_*(*b_i_*) were recorded (see Tables 4 and 5).

It is conceivable that in the optimal parameter set *θ*_0_, the low sensitivity of specific parameters is a result of the local optimization procedure. We checked that this was not the case for each low-sensitivity parameter *k* by calculating the cost function *E*(*θ′*) where *θ′ = θ*_0_, except for *k* whose value was taken 10% smaller (and 10% larger) than its optimal value. We verified that in all cases *E*(*θ′*)≈*E*(*θ*_0_).

## Supporting Information

Text S1 Functional significance of nonlinear mechanisms and sensitivity analysis of model parameters.
(0.08 MB DOC)
