# Supplementary material for: Computational Model of the Insect Pheromone Transduction Cascade
Source: PLoS Comput Biol. 2009 Mar 20;5(3):e1000321. doi: 10.1371/journal.pcbi.1000321 (PMC2649447; doi:10.1371/journal.pcbi.1000321)
Supplement: Text S1 — Functional significance of nonlinear mechanisms and sensitivity analysis of model parameters. (0.08 MB DOC) [file pcbi.1000321.s001.doc]

SUPPLEMENTARY TEXT

to accompany *Model of the Insect Pheromone Transduction Cascade*

Yuqiao GU^1^, Philippe LUCAS^1^ and Jean-Pierre ROSPARS^1,2^

^1^ INRA, UMR 1272 Physiologie de l'insecte: Signalisation et Communication, F-78000 Versailles

^2^ INRA, Mathématiques et Informatique appliquées, F-78352 Jouy-en-Josas

Contents

1.Functional significance of nonlinear mechanisms (with Fig. S1)

2. Sensitivity analysis of model parameters (with Tables S1-S3)

**1.Functional significance of nonlinear mechanisms**

In the biochemical and electrical network simulated here, several nonlinear, especially feedback, mechanisms are involved. Their functional role can be revealed by removing them one by one from the model and observing the effects on the height, rising time and falling time of the receptor potential or its experimentally recorded counterpart, the sensillar potential SP. When the feedback inhibition of PKC* on the production of second messengers (step 16 in **Fig. 3**) is removed (**Fig. S1**, dashed lines, “E*”), the computed SP is almost the same as the normal one (dotted line, “*SP*_exp_”) at low uptakes. However, for higher uptakes its amplitude becomes much higher and falling times become much longer, whereas the rising time is much longer only at intermediate uptakes (maximum at 10^-3^ µM/s). The strength of the feedback inhibition effect on the effector enzyme depends obviously on the respective values of PKC* concentration with respect to the IC_50_ (*K*_is_): at the lowest uptake (10^-4.75^ µM/s) *PKC** is much smaller than *K*_is_ (the ratio *K*_is_/max(*PKC**) is 91), whereas at the highest uptake (31.6 µM/s) *PKC** is much larger than *K*_is_ (the ratio max(*PKC**)/*K*_is_ is 66).

Similar effects are produced by removing the CaCaM (steps 11, 12 in **Fig. 3**) or PKC* feedback inhibition on the second-messenger-gated channels (**Fig. S1**, solid lines, “dep cur”), except that they appear at lower uptakes. For example, the maximum rising time is now at 10^-4.75^ µM/s and returns to normal above 10^-3.5^ µM/s. The inhibition strength of the second-messenger-gated channels varies with uptake as that of the effector enzyme. For example, for the cationic current the ratio *K*_icat_/max(*CaCaM*) decreases from 8.5 to 2.5×10^-2^. However, as previously noted, the PKC* feedback on Cl^-^ conductance is practically negligible at all uptakes (**Fig. 5B**).

In a system with a constant conductance of the Ca^2+^ extrusion current the height of the SP is higher than normal at low uptakes and its rising time higher at low uptakes and lower at intermediate uptakes (**Fig. S1**, “NCX”).

**
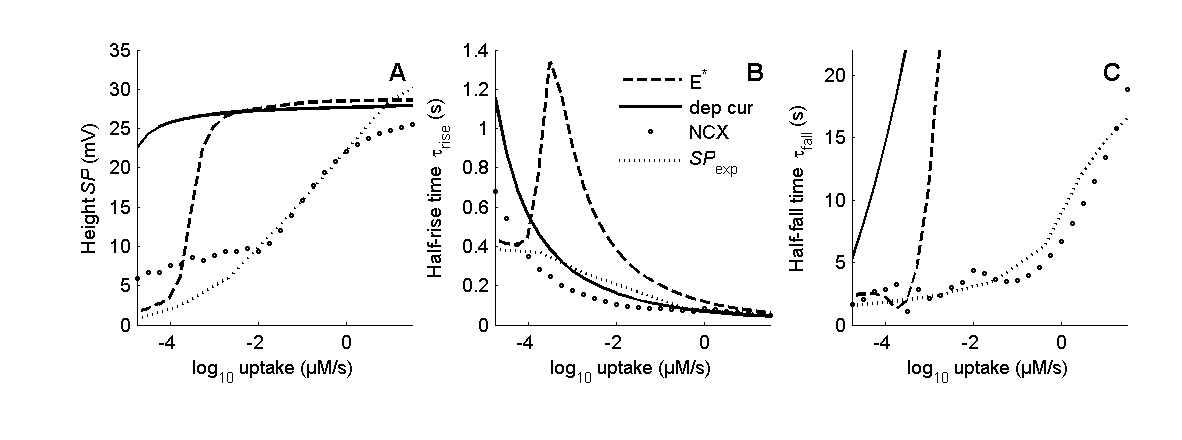
**

**Fig. S1**. Comparison of dose-response characteristic of SP (height in A, rising time in B and falling time in C) with one of the nonlinear mechanisms removed: no PKC* inhibition of E* (linear second messenger production rate; “E*”, dashed line); no inhibition of the three depolarizing currents due to IP_3_-gated channels, DAG- gated channels and Ca^2+^-gated Cl^-^ channels (“dep cur”, solid); linear Na^+^/Ca^2+^ exchange currents (“NCX”, circles). The curves diverge most from the experimentally measured SP characteristics (“SP_exp_”, dotted) at different pheromone uptakes.

**2. Sensitivity analysis of model parameters**

The sensitivity of the system to the parameters controlling each biochemical and electrical step was analyzed. The relative central finite difference sensitivity of the three dose-response characteristics -- height, half-rise time and half-fall time -- of the sensillar potential SP (model output) at all uptakes was calculated using *S*_r_*_u_*(*b*_i_), see eq. (60) in main text, for each of the 38 fitted parameters *b_i_*. The largest absolute value *S*_r_(*b*_i_) of the *S*_r_*_u_*(*b*_i_) over all uptakes was determined (here it is called “relative sensitivity” *S*_r_ for short). The analysis of these relative sensitivities (114 values in all) led to the following conclusions:

First, each parameter has its greatest influence on one of the characteristics (see **Tables 4 and 5** in main text). The most influenced characteristic is usually the falling time (63%), less frequently the rising time (21%) and the height (16%). Interestingly, 88% of the corresponding uptakes for falling time are in the upper range (log*U* > -1.75), whereas 83% of the uptakes for height and 88% for rising time are in the lower range (log*U* ≤ -3.75). This means that *the determination of parameter values depends mostly on height and rising time at low uptakes, and on falling time at high uptakes*.

Second, the parameters can be classified in four categories according to the largest relative sensitivity over all three characteristics. (i) For 9 parameters (*s*_M_, *n*_iCa_*, k*_cc2_, *K*_mCl_, *i*_Mcat_, *A*_k_*, i*_MCa_, *i*_MCl_ and *n*_iCl_) it is less than 3%. These are the least well determined parameters. (ii) For 9 parameters (*k*_pd2_, *G*_MK_, *f*_Ca_, *k*_s2_, *K*_MCa_, *G*_MCl_, *E*_Cl_, *K*_iCa_, *k*_ap2_) it is greater than 3% and less than 10%. (iii) For 9 parameters (*E*_x_, *K*_iCl_, *k*_ap1_, *n*_Ca_, *f*_cat_, *k*_cc1_, *K*_mx_, *n*_icat_, *n*_is_) it is greater than 10% and less than 100%. (iv) Finally for the last 11 parameters (*G*_Mcat_, *n*_x_, *n*_Cl_, *G*_MCa_, *n*_cat_, *K*_pd_, *K*_icat_, *K*_mcat_, *G*_Mx_, *K*_k_, *K*_is_,) it is greater than 100%. These are the best determined parameters.

**Tables S1-S3** list separately the most and least influential parameters on height, half-rise time and half-fall time. To build these tables the 3×38 relative sensitivities *S*_r_ over all characteristics where divided in 3 classes of almost the same size, *S*_r_< 2.5% (37 occurrences), 2.5% < *S*_r_< 50% (40) and *S*_r_> 50% (37). Finally, modifying the three degradation and dissociation rate parameters (*k*_cc2_, *k*_ap2_, *k*_pd2_) is equivalent to the opposite modification of the corresponding activation rate parameters. However the response characteristics are more sensitive to the activation than to the degradation rate parameters. The effect on SP of parameters controlling the feedback inhibition of the Cl^-^ current (when added to the model) is negligible.

**Table S1.** Most and least sensitive parameters controlling heights ^1^

----------------------------------------------------------------------------

Not sensitive Sensitive Very sensitive

*S*_r_ < 0.025 0.025 < *S*_r_ < 0.5 *S*_r_  > 0.5

----------------------------------------------------------------------------

IP_3_-DAG metabolism (*s*_M_) Cl^-^ channel (*G*_MCl_) Ca+CaM association (*k*_cc1_)

K^+^ channel (*A*_K_) IP_3_-DAG metabolism (*k*_s2_) cationic channel (*n*_icat_)

Ca^2+^ channel (*i*_MCa_) Ca^2+^ channel (*K*_iCa_) cationic channel (*G*_Mcat_)

Ca^2+^ channel (*n*_iCa_) CaPKCDAG dissociation (*k*_ap2_) Cl^-^ channel (*n*_Cl_)

Cl^-^ channel (*K*_mCl_) Cl^-^ channel (*K*_iCl_) cationic channel (*n*_cat_)

CaCaM dissociation (*k*_cc2_) Ca+PKCDAG association (*k*_ap1_) PKC+DAG association (*k*_pd1_)

Ca^2+^ factor (*f*_Ca_) Ca^2+^ channel (*n*_Ca_) cationic channel (*K*_icat_)

Cl^-^ channel (*E*_Cl_) Ca^2+^ extrusion (*K*_mx_) cationic channel (*K*_mcat_)

cationic channel (*i*_Mcat_) cationic factor (*f*_cat_) K^+^ channel (*K*_K_)

Cl^-^ channel (*i*_MCl_) Ca^2+^ extrusion (*n*_x_) Ca^2+^ extrusion (*G*_Mx_)

Ca^2+^ extrusion (*E*_x_) PKC inhibition (*n*_is_) PKC inhibition (*K*_is_)

Cl^-^ channel (*n*_iCl_) Ca^2+^ channel (*G*_MCa_)

K^+^ channel (*G*_MK_)

Ca^2+^ channel (*K*_mCa_)

PKCDAG dissociation (*k*_pd2_)

----------------------------------------------------------------------------

^1^ Parameters listed in order of increasing influence.

**Table S2.** Most and least sensitive parameters controlling rising times ^1^

---------------------------------------------------------------------------

Not sensitive Sensitive Very sensitive

*S*_r_ < 0.025 0.025 < *S*_r_ < 0.5 *S*_r_  > 0.5

---------------------------------------------------------------------------

IP_3_-DAG metabolism (*s*_M_) Ca^2+^ channel (*K*_iCa_) Ca^2+^ extrusion (*K*_mx_)

Ca channel (*i*_MCa_) IP_3_-DAG metabolism (*k*_s2_) cationic channel (*n*_icat_)

cationic channel (*i*_Mcat_) K^+^ channel (*G*_MK_) cationic channel (*n*_cat_)

Ca^2+^ channel (*n*_iCa_) Ca^2+^ factor (*f*_Ca_) Cl^-^ channel (*n*_Cl_)

Cl^-^ channel (*K*_mCl_) Ca^2+^ channel (*K*_mCa_) Ca^2+^ extrusion (*n*_x_)

CaCaM dissociation (*k*_cc2_) CaPKCDAG dissociation (*k*_ap2_) Ca^2+^ channel (*G*_MCa_)

K^+^ channel (*A*_K_) Ca+PKCDAG association (*k*_ap1_) PKC+DAG association (*k*_pd1_)

Cl^-^ channel (*i*_MCl_) Ca^2+^ channel (*n*_Ca_) cationic channel (*K*_icat_)

Ca^2+^ extrusion (*E*_x_) Cl^-^ channel (*K*_iCl_) cationic channel (*K*_mcat_)

Cl^-^ channel (*n*_iCl_) cationic factor (*f*_cat_) Ca^2+^ extrusion (*G*_mx_)

PKC DAG dissociation (*k*_pd2_) PKC inhibition (*n*_is_) K^+^ channel (*K*_K_)

Cl^-^ channel (*G*_MCl_) Ca+CaM association (*k*_cc1_) PKC inhibition (*K*_is_)

Cl^-^ channel (*E*_Cl_) cationic channel (*G*_Mcat_)

---------------------------------------------------------------------------

^1^ Same presentation as in Table S1.

**Table S3.** Most and least sensitive parameters controlling falling times ^1^

---------------------------------------------------------------------------

Not sensitive Sensitive Very sensitive

*S*_r_ < 0.025 0.025 < *S*_r_ < 0.5 *S*_r_  > 0.5

---------------------------------------------------------------------------

IP_3_-DAG metabolism (*s*_M_) Cl^-^ channel (*i*_MCl_) Ca^2+^ extrusion (*K*_mx_)

CaCaM dissociation (*k*_cc2_) K^+^ channel (*G*_MK_) Ca^2+^ channel (*G*_MCa_)

Ca^2+^ channel (*n*_iCa_) Cl^-^ channel (*n*_iCl_) cationic channel (*n*_icat_)

Cl^-^ channel (*K*_mCl_) PKC DAG dissociation (*k*_pd2_) Ca^2+^ extrusion (*n*_x_)

Ca^2+^ factor (*f*_Ca_) Ca^2+^ channel (*K*_mCa_) Cl^-^ channel (*n*_Cl_)

cationic channel (*i*_Mcat_) Cl^-^ channel (*G*_MCl_) PKC inhibition (*n*_is_)

K^+^ channel (*A*_K_) Cl^-^ channel (*E*_Cl_) cationic channel (*G*_Mcat_)

Ca^2+^ channel (*i*_MCa_) Ca^2+^ channel (*K*_iCa_) cationic channel (*K*_icat_)

IP_3_-DAG metabolism (*k*_s2_) CaPKCDAG dissociation (*k*_ap2_) cationic channel (*n*_cat_)

Ca^2+^ extrusion (*E*_x_) PKC+DAG association (*k*_pd1_)

Cl^-^ channel (*K*_iCl_) cationic channel (*K*_mcat_)

Ca+CaM association (*k*_cc1_) Ca^2+^ extrusion (*G*_mx_)

Ca+PKCDAG association (*k*_ap1_) K^+^ channel (*K*_K_)

Ca^2+^ channel (*n*_Ca_) PKC inhibition (*K*_is_)

cationic factor (*f*_cat_)

---------------------------------------------------------------------------

^1^ Same presentation as in Table S1.
